# Modelling the influence of vitamin D and probiotics on inflammation and the intestinal microbiota in older adults

**DOI:** 10.1038/s41598-025-26132-8

**Published:** 2025-11-26

**Authors:** Susan Franks, Joanne Dunster, Simon Carding, Janet Lord, Martin Hewison, Philip Calder, John King

**Affiliations:** 1 School of Mathematical Sciences, University of Nottingham, Nottingham, UK; 2 Institute for Cardiovascular and Metabolic Research, University of Reading, Reading, UK; 3 Quadram Institute Bioscience, Norwich, UK; 4 Norwich Medical School, University of East Anglia, Norwich, UK; 5 Institute of Inflammation and Ageing, University of Birmingham, Birmingham, UK; 6 Institute of Metabolism and Systems Research, University of Birmingham, Birmingham, UK; 7 School of Human Development and Health, Faculty of Medicine , University of Southampton, Southampton, UK; 8 NIHR Southampton Biomedical Research Centre, University Hospital Southampton NHS Foundation Trust and University of Southampton, Southampton, UK

**Keywords:** Computational biology and bioinformatics, Immunology, Microbiology, Mathematics and computing

## Abstract

The relationship between the intestinal microbiota and human health during ageing is an area of increasing interest due to increasing health challenges experienced by ageing populations. This paper develops a mathematical model describing the age-related biological changes associated with alterations to the microbiota, vitamin D levels, immunosenescence and inflammageing to determine the likely impact of manipulating the intestinal microbiota with dietary components. Age-dependent parameters are incorporated into a previously developed model to determine the evolution of intestinal bacterial populations, vitamin D receptor:1,25 dihydroxyvitamin D levels, epithelial barrier integrity and immune response with increasing age. Results suggest an age-related decline in both innate and adaptive immunity, weakening of the intestinal barrier, elevation in systemic inflammation and reduced serum vitamin D, resulting in individuals over 60 years old becoming vitamin D deficient (<50 nmol/L). Numerical simulations indicate that administration of probiotics and/or vitamin D supplements upregulates the VDR complex at all ages, which helps restore epithelial barrier function, particularly in older adults in whom the intestinal barrier has been compromised. The greatest benefit is derived from co-supplementation with probiotics and age-dependent doses of vitamin D. Finally, the value of gathering additional experimental data motivated by the modelling insights is discussed.

## Introduction

The number and proportion of older people are increasing in the UK and many other countries as people are living longer. However, while lifespan is increasing, healthspan is not keeping pace and many older people live with significant illness for many years. Ageing ultimately leads to the loss of functional capacity in many body systems, including the cardiovascular, musculoskeletal, osteoarticular and neuroendocrine. A hallmark feature of ageing is immune decline, termed immunosenescence, with the functional decline of the innate and adaptive immune systems resulting in compromised immunity^1–3^. Paradoxically, in parallel there is an elevation in systemic inflammation with ageing, a phenomenon termed inflammageing^4,5^. Inflammageing and its associated conditions, including cardiovascular disease, metabolic diseases, sarcopenia and osteoporosis, some cancers and possibly dementia, make significant contributions to morbidity, poor quality of life, increased social and health care costs, and mortality in older people. Furthermore, immunosenescence increases susceptibility to infections, contributing to illness and mortality^6^. Importantly, these immune changes also limit responses to public health measures such as influenza vaccination, where average efficacy of vaccines declines from 80-90% in younger populations to 30-50% in older adults^7,8^. Thus, there is an urgent need to understand and overcome the drivers of age-related immune changes that cause ill health.

The intestinal microbiota has an influence on host health by, for example, acting through direct and indirect effects on the development and function of the host immune system and inflammatory response^9,10^. The structural complexity and functional capability of the intestinal microbiota also decline with ageing^11–14^. How this occurs, and the nature of the relationship between changes in the microbiota, the onset of low-grade inflammation, declining gastrointestinal tract function and immunosenescence in older people, is not well described. The loss of intestinal barrier function in older people, which can result in translocation of bacterial endotoxins and whole bacteria into the bloodstream, is a plausible link between intestinal dysbiosis and inflammageing, and is consistent with the concept that manipulation of the intestinal microbiota may be of therapeutic benefit in older people^15^.

Nutritional approaches may be used to modify the intestinal microbiota^16,17^, and high dose vitamin D supplements have been shown to alter the faecal microbiota both in animal models^18^ and in humans^19,20^. Sufficient vitamin D has also been shown to support the immune system, maintaining innate and cell-mediated immunity and regulating inflammation^21–28^, suggesting a possible link between vitamin D levels, the microbiota and inflammatory status. Vitamin D levels are generally low in the UK population^29^, particularly in older people, and are inversely associated with infection risk^30^. There is therefore a potential role of vitamin D in reversing immunosenescence and inflammageing that requires further exploration.

The intestinal microbiota may also be manipulated through oral intake of probiotics. Probiotic organisms, for example *Lactobacillus plantarum*, have been shown to survive passage through the gastrointestinal tract and to colonise the colon in humans. The TIFN101 strain has been shown to affect the immune system positively in humans (although it has yet to be investigated in older adults) by supporting the maintenance of immune cells^31^ and stimulating memory responses and antigen presentation^32^, supporting intestinal barrier function^33^ and protecting against intestinal inflammation by eliciting inflammatory signalling molecules^34^. Similarly, researchers who conducted a study in an older Japanese cohort with a similar probiotic, *L. plantarum* OLL2712, concluded that ingestion has protective effects against immune memory function decline in older adults and observed a decrease in certain bacteria associated with inflammation^35^. There is therefore the potential for supplementation to benefit older individuals in whom there are changes in the intestinal microbiota^11–14^, immune decline^1–3^ and elevated inflammation^4,5^.

The interactions between the microbiome, vitamin D status and the immune response are complex and Franks *et al*.^36^ presented a novel mathematical model to understand their relationship and effects of individual and co-supplementation of vitamin D and probiotics. However, there is an essential need to understand the age-related biological changes associated with changes to the microbiota, low vitamin D levels, immunosenescence and inflammageing, and to assess the potential benefits of modifying the intestinal microbiota and regulating inflammation with probiotics and vitamin D supplements. Hence, the aim of this paper is to adapt the model proposed in^36^ to account for the effect of ageing on the microbiome and immune response. In the Methods section, the age-related biological changes in the microbiota, vitamin D and its metabolites and the epithelial barrier and immune response are discussed and how parameters within these models alter with age. The complete model is solved numerically to assess the impact of ageing in the Results section, and whether these changes are reversible with vitamin D supplements only, probiotics only and co-supplementation is then examined.

**Fig. 1:**
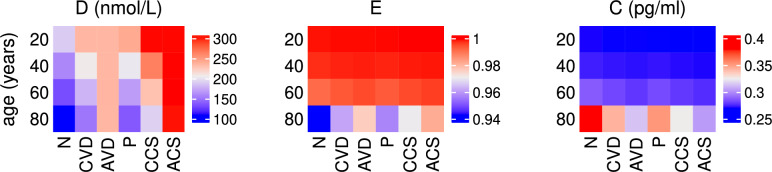
Effect of supplementation on 25(OH)D, epithelial lining and pro-inflammatory cytokines with age. The predicted steady state values on day 200 of the concentration of extracellular 25(OH)D (D), volume fraction of healthy cells in the epithelium (E) and concentration of pro-inflammatory cytokines (C) with age. A daily intervention is administered from day 0 to day 200 to individuals aged 20-80 years old for dosing regimens N (no supplementation), CVD (a constant daily dose of vitamin D), AVD (an age-dependent daily dose of vitamin D), P (a constant daily dose of probiotics), CCS (a combined constant daily dose of vitamin D and probiotics) and ACS (a combined age-dependent daily dose of vitamin D and constant daily dose of probiotics).

In order to illustrate the types of insight for which the current modelling strives, Fig. 1 demonstrates the beneficial outcomes of these supplementation regimens on the serum levels of vitamin D, healthy intestinal lining and pro-inflammatory cytokine concentration with age. An increase in vitamin D upregulates the VDR complex which helps repair the epithelial barrier function and reduces inflammation.

**Fig. 2:**
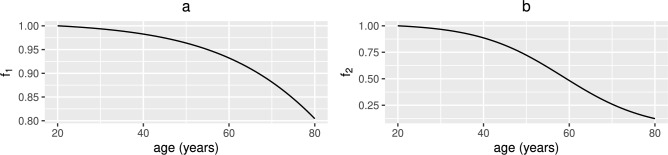
Functional forms for $f_{1}(t)$ and $f_{2}(t)$. (a) The assumed decline of available macro and micronutrients with increasing age, $f_{1}(t)=1/(1+10^{-5.9+0.06\text {age}})+0.008$, and (b) the decline of pathogenic elimination by reduced autophagy, bacteria clearance, secretory IgA and SCFAs, $f_{2}(t)=1(1+10^{-5.9+0.1\text {age}})+0.02$ where age=(t+7300)/365 years and $0\le t\le 21900$ days.

## Results

### Ageing model

**Table 1:** Definition, value and units of the nutrient model parameters taken from^36^.

Parameter	Description	Value, Units & Source
$N_{ma}^{0}$	Rate of intake of macronutrients	400 g/day^45^
$N_{mi}^{0}$	Rate of intake of micronutrients	9 g/day^45^
*q*	Rate of faecal removal of excess nutrients and bacteria	0.13 day^-1^^65^
$F^{0}$	Rate of intake of commensal bacteria	$\mathrm {1.24\times 10}^{9}$ CFU/day^66^
$P^{0}$	Rate of intake of pathogenic bacteria	$\mathrm {0.06\times 10}^{9}$ CFU/day^66^
$P_{b}$	Rate of intake of probiotics	$\mathrm {1\times 10}^{9}$-$\mathrm {1\times 10}^{11}$ CFU/day
*K*	Carrying capacity	$\mathrm {1\times 10}^{14}$ CFU
$\eta _{1}$	Rate of uptake of macronutrients by commensal bacteria	$\mathrm {1\times 10}^{-14}$ (CFU day)^-1^
$\eta _{2}$	Rate of release of micronutrients from degradation of pathogenic bacteria	$\mathrm {1\times 10}^{-17}$ (CFU day)^-1^
$\eta _{3}$	Rate of consumption of micronutrients by commensal bacteria	$\mathrm {1\times 10}^{-14}$ (CFU day)^-1^
$\eta _{4}$	Rate of uptake of micronutrients by pathogenic bacteria	$\mathrm {1\times 10}^{-14}$ (CFU day)^-1^
$\eta _{5}$	Rate of consumption of micronutrients by host epithelial cells	0.01 day^-1^
$\eta _{6}$	Rate of utilisation of metabolites by epithelial cells	0.1 day^-1^
$\eta _{7}$	Rate of utilisation of metabolites by commensal bacteria	$\mathrm {1\times 10}^{-17}$ (CFU day)^-1^
$\eta _{8}$	Rate of production of alternate nutrients	$\mathrm {2.59\times 10}^{-9}$ ml/(ng CFU day)
$\eta _{9}$	Rate of consumption of alternate nutrients by pathogens	$\mathrm {1\times 10}^{-14}$ (CFU day)^-1^
$\beta _{1}$	Proportionality parameter	$\mathrm {2.44\times 10}^{5}$ CFU/ng
$\beta _{2}$	Proportionality parameter	$\mathrm {2.44\times 10}^{4}$ CFU/ng
$\beta _{3}$	Rate at which pathogenic bacteria are destroyed by autophagy and AMPs from epithelial cells	0.5 day^-1^
$\beta _{4}$	Rate at which pathogens are destroyed by macrophages	$\mathrm {9.17\times 10}^{-9}$ ml/day
$\beta _{5}$	Rate at which pathogens are destroyed by commensals	$\mathrm {1\times 10}^{-17}$ (CFU day)^-1^
$\beta _{6}$	Rate at which pathogens are destroyed by sIgA	$\mathrm {1.03\times 10}^{-6}$ ml/day

In developed countries, adults consume on average approximately 400 g/day of macronutrients and 9 g/day of micronutrients^45^. They typically expel 128 g/day of faeces of which there are approximately 1$\times 10^{11}$ bacteria/g of wet stool so that the total number of bacteria removed in the faeces is 1.28$\times 10^{13}$ bacteria/$\hbox {day}$^65^. Expressing this in terms of the total number of bacteria in the intestine gives an approximate value of $q=0.13$ $\hbox {day}^{-1}$. In a healthy diet we consume approximately 1.3$\times 10^{9}$ CFU/day^66^ and we assume 5% of these microbes are pathogenic. There are approximately 1$\times 10^{14}$ CFU of bacteria in the intestinal tract so we assume that the carrying capacity *K* equals this value.

**Table 2:** Definition, value and units of the vitamin D model parameters taken from^36^.

Parameter	Description	Value Units & Source
$D^{0}$	Production of 25(OH)D from diet and sunlight	variable nM/day
$k_{d}/\delta$	Maximal rate of conversion of extracellular 25(OH)D to 1,25$\text {(OH)}_{2}$D	24 nM/day^67^
$K_{D}$	Michaelis Menten constant for extracellular 25(OH)D binding to CYP27B1	1000 nM^67^
$\delta _{1}$	$D^{0}\delta _{1}$ is the maximum production rate of vitamin D dependent upon probiotics	0.3
$\delta _{2}$	Degradation of extracellular 25(OH)D	0.048 $\hbox {day}^{-1}$^68^
$\delta _{3}$	Degradation of extracellular 1,25$\text {(OH)}_{2}$D	14.4 $\hbox {day}^{-1}$^68^
$\mu _{f}$	Proportion of total extracellular 25(OH)D that is free	0.05 %^67^
$\mu _{a_{f}}$	Proportion of total extracellular 1,25$\text {(OH)}_{2}$D that is free	0.85 %^67^
$\sigma _{1}$	Permeability of macrophages to free 25(OH)D or 1,25$\text {(OH)}_{2}$D	144 $\hbox {day}^{-1}$^67^
$\sigma _{2}$	Permeability of epithelial cells to free 25(OH)D or 1,25$\text {(OH)}_{2}$D	144 $\hbox {day}^{-1}$
$k_{d_{i}}/\delta$	Maximal rate of conversion of intracellular 25(OH)D to 1,25$\text {(OH)}_{2}$D	24 nM/day^67^
$K_{D_{i}}$	Michaelis Menten constant for intracellular 25(OH)D binding to CYP27B1	1000 nM^67^
$\delta _{4}$	Degradation of intracellular 25(OH)D	0.048 $\hbox {day}^{-1}$^68^
$\delta _{5}$	Degradation of intracellular 1,25$\text {(OH)}_{2}$D	14.4 $\hbox {day}^{-1}$^68^
$\delta _{6}$	Rate at which 1,25$\text {(OH)}_{2}$D binds to VDR	24$\times 10^{-7}$ $\hbox {nM}^{-1}$ $\hbox {day}^{-1}$^67^
$\delta _{7}$	Rate of degradation of VDR:1,25$\text {(OH)}_{2}$D	0.024 $\hbox {day}^{-1}$
$\delta _{8}a/K_{V}$	Concentration of VDR	1.2 nM^67^
$K_{V}$	Saturation constant	1 CFU/day
$K_{\delta }$	Saturation constant	5$\times 10^{8}$ CFU/day

**Fig. 3:**
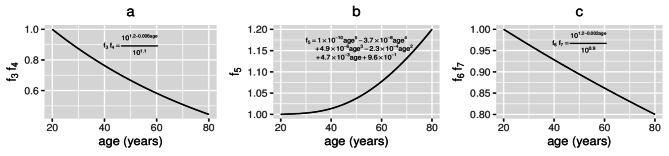
Functional forms for $f_{3}(t)$, $f_{4}(t)$, $f_{5}(t)$, $f_{6}(t)$ and $f_{7}(t)$. The assumed age-related changes of (**a**) vitamin D production ($f_{3}(t)$) and renal function ($f_{4}(t)$) where $f_{3}=f_{4}=10^{0.1-0.006\text {age}}$, (**b**) macrophage and epithelial cell membrane permeability ($f_{5}(t)=1\times 10^{-10}\text {age}^{5}-3.7\times 10^{-8}\text {age}^{4}+4.9\times 10^{-6}\text {age}^{3}-2.3\times 10^{-4}\text {age}^{2}+4.7\times 10^{-3}\text {age}$ $+9.6\times 10^{-1}$) and (**c**) metabolism of intracellular 25(OH)D to 1,25(OH)_2_D ($f_{6}(t)$) and VDR:1,25(OH)_2_D binding ($f_{7}(t)$) where $f_{6}=f_{7}=10^{0.3-0.002\text {age}}$. As in Fig. 2, age=(t+7300)/365 years and $0\le t\le 21900$ days.

**Fig. 4:**
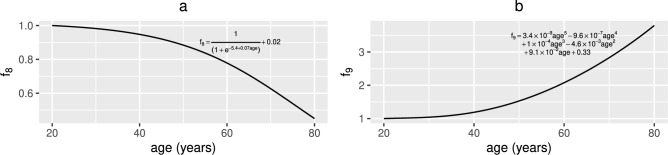
Functional forms for $f_{8}(t)$ and $f_{9}(t)$. (**a**) The assumed age-related decline of epithelial cell proliferation, reparation and macrophage clearance of damaged epithelial cells ($f_{8}(t)=1/(1+10^{-5.4+0.07\text {age}})+0.02$) and (**b**) the increase in damage to healthy epithelial cells due to the mucosal layer becoming less effective at protecting them ($f_{9}(t)=3.4\times 10^{-9}\text {age}^{5}-9.6\times 10^{-7}\text {age}^{4}+1\times 10^{-4}\text {age}^{3}-4.6\times 10^{-3}\text {age}^{2}+9.1\times 10^{-2}\text {age}$ $+0.33$) where age=(t+7300)/365 years and $0\le t\le 21900$ days.

**Fig. 5:**
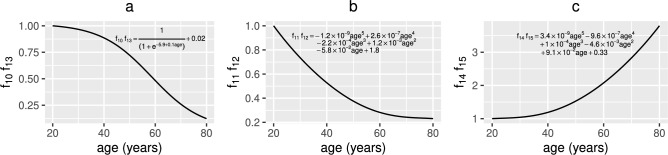
Functional forms for $f_{10}(t)$, $f_{11}(t)$, $f_{12}(t)$, $f_{13}(t)$, $f_{14}(t)$ and $f_{15}(t)$. (**a**) The assumed age-related decline of macrophage activation by pro-inflammatory cytokines ($f_{10}(t)$) and activation and production of anti-inflammatory cytokines by macrophages ($f_{13}(t)$) where $f_{10}=f_{13}=1/(1+10^{-5.9+0.1\text {age}}+0.02$. (**b**) The age-related decline in T-cell ($f_{11}(t)$) and B-cell ($f_{12}(t)$) proliferation where $f_{11}=f_{12}=-1.2\times 10^{-9}\text {age}^{5}+$ $2.6\times 10^{-7}\text {age}^{4}-2.2\times 10^{-4}\text {age}^{3}+1.2\times 10^{-3}\text {age}^{2}-5.8\times 10^{-2}\text {age}+1.8$. (**c**) The increase with age of production of pro-inflammatory cytokines by macrophages ($f_{14}(t)$) and T-cells ($f_{15}(t)$) where $f_{14}=f_{15}=3.4\times 10^{-9}\text {age}^{5}-9.6\times 10^{-7}\text {age}^{4}+ 1\times 10^{-4}\text {age}^{3}$ $-4.6\times 10^{-3}\text {age}^{2}+9.1\times 10^{-2}\text {age}+0.33$. As in previous figures, age=(t+7300)/365 years and $0\le t\le 21900$ days.

Equations (3)–(23) were solved numerically to large time using the baseline parameter values in Tables 1, 2 and 3 and the value of the functions represented by Figs. 2, 3, 4 and 5 at age 20 years. It was assumed that microbiota consist mainly of commensal (beneficial) bacteria and the epithelial barrier is healthy i.e.

1 $$\begin{aligned} F_{0}=0.99\times 10^{14}, \ \ P_{0}=0.01\times 10^{14}, \ \ E_{0}=1. \end{aligned}$$

All the remaining initial conditions were zero. When steady state had been attained, these values of the nutrient concentrations, bacterial populations, the concentration of vitamin D and its metabolites, volume fraction of healthy and damaged epithelial cells, immune cell densities and concentrations of pro- and anti-inflammatory mediators for a healthy young adult were used as the new initial conditions denoted by subscript _ss_ in Eqs. (9), (15) and (24). The system of equations was then solved from 20 to 80 years (i.e. from $t=0$ to 21900 days) to determine how the variables change within a lifetime. The ODEs were solved using the ode solver in R with the default integrator lsoda (a discussion on the use of the lsoda solver is provided in the supplementary material). The code, in the form of a *R* notebook, is available in the supplementary material in^36^. However, the age-dependent functions $f_{1}(t)-f_{15}(t)$ would need to be added into the governing equations.

Thresholds for vitamin D deficiency in the UK currently differ from those defined elsewhere (termed World in this paper). As outlined in^39^, the UK defines those with serum levels less than 25 nmol/L having an increased risk of vitamin D deficiency compared with levels less than 50 nmol/L in the majority of other countries^40^. Vitamin D levels may be inadequate (or insufficient) in the UK when serum 25(OH)D is between 25 and 50 nmol/L (compared to levels between 50 and 75 nmol/L^40^) and vitamin D levels are sufficient for most people when serum 25(OH)D is greater than 50 nmol/L (compared to levels greater than 75 nmol/L elsewhere^40^).

Figures 7 and 8 therefore present model predictions for when a young person, age 20 years, has adequate levels of 25(OH)D as provided by the two threshold definitions i.e. 50 nmol/L and 75 nmol/L. Note that intake levels of 25(OH)D from sunlight $D^{0}$ and dietary sources $D^{1}$ in the latter case need to be much greater to attain the higher concentration of serum 25(OH)D i.e. 5.3 nmol/L day^−1^ compared with 3.55 nmol/L day^−1^. Here $P_{b}=0$ as there is no probiotic supplementation.

Figure 7a and b indicate that the concentration of macronutrients and micronutrients decreases with age as the absorption of nutrients from food declines. The metabolite concentration (Fig 7c) also decreases as commensal bacteria have less macronutrients to convert by fermentation into metabolites, thereby resulting in less fuel being available to intestinal epithelial cells, instead favouring conversion to alternate nutrients by pathogenic (pro-inflammatory) bacteria (Fig 7d). This results in an increase in pathogenic bacteria, and a corresponding decrease in commensals (Fig. 7f and e, respectively), as elimination of pathogens by SCFAs, AMPs, autophagy and sIgA produced by commensals, epithelial cells, macrophages and B cells is reduced with age. There is an increase in non-viable and damaged epithelial cells (Fig. 8b) (and a corresponding decrease in healthy epithelial cells (Fig. 8a) due, in part, to a decrease in metabolites providing energy for their proliferation) with damaged epithelial cells being under stress from the invading pathogens, increasing signalling of pro-inflammatory cytokines (Fig. 8h) and inhibiting anti-inflammatory cytokines (Fig. 8g) which stimulate macrophages, plasma B-cells and T-cells. The production of pro-inflammatory cytokines by macrophages and T-cells also increases with age. The increased inflammatory environment allows the enhanced conversion of metabolic byproducts generated by commensal bacteria into alternate nutrients, thereby further fuelling the proliferation of pathogenic bacteria which downregulates the production of regulatory cells (Fig. 8d). Newly arriving macrophages have a more pro-inflammatory phenotype in older adults that is reduced under the effect of anti-inflammatory mediators. However, Fig. 8c shows that the overall macrophage density is reduced, due mainly to the increased senescence-associated phenotype (reduced proliferation). A decline in the T-cell and plasma B-cell densities between age 20 and 65 years is also observed (Fig. 8e and f, respectively) as the thymic involution, and a skewing in the bone marrow towards non-lymphoid cells, leads to a decreased output of naïve T-cells and the activation and maturation of B-cells required to make antibodies reduces with age. However, due to the increasing epithelial barrier disintegration in individuals >65 years, an increase in T- and plasma B-cells is observed potentially due to activation of the immune system in this age group.

Figure 9a–e show a decrease in the concentration of 25(OH)D and its metabolites with age due to a decline in the generation and metabolism of 25(OH)D and binding of the VDR:1,25(OH)_2_D complex. It has been assumed that a 20 year old individual has healthy levels of 25(OH)D i.e. 50 nmol/L or 75 nmol/L based on the threshold definitions discussed above for the UK and the World, respectively. In the UK scenario, all individuals over 20 years have insufficient levels of 25(OH)D i.e. between 25 and 50 nmol/L but no individual under 80 years becomes deficient. Using the higher threshold definitions, individuals aged over 20 and under 66 years have insufficient levels i.e. between 50 and 75 nmol/L and those over 66 years are deficient. It therefore takes longer to reach UK defined deficiency levels, but for all ages there is increased inflammation and damage to the epithelial intestinal lining. Figure 9a–e indicate that the difference in the concentration of serum 25(OH)D and its metabolites between the UK and World definitions at age 20 is greater than that at age 80 due to there being more available to be metabolised. This is also observed for the concentration of anti-inflammatory cytokines due to its dependence upon the concentration of VDR:1,25$\text {(OH)}_{2}$D complex. Conversely, the difference between the concentration of alternate nutrients, volume fraction of healthy epithelial cells, density of macrophages and concentration of pro-inflammatory cytokines increases for older individuals.

A 50% decline in extracellular 25(OH)D results in approximately a 75% decrease in extracellular 1,25(OH)_2_D. This is due to the reduced generation of vitamin D in the skin and the decline in renal function with age. A decrease in VDR:1,25(OH)_2_D increases the activation of macrophages as it upregulates the pro-inflammatory cytokines but reduces the production of anti-inflammatory mediators by epithelial cells. Macrophages produce anti-inflammatory mediators after consuming damaged epithelial cells and pathogens, but phagocytosis is decreased in older adults so an overall decline in the concentration of anti-inflammatory cytokines is observed. The density of regulatory cells decreases as pathogenic bacteria downregulate their production.

A table comparing key model outputs to the literature is provided in Table 4.

#### Global sensitivity analysis

Figure 10 shows the parameters that most strongly influence the predicted pathogenic population, volume fraction of healthy epithelial cells and concentration of pro-inflammatory cytokines, larger absolute values corresponding to greater influence. The most influential parameters in the model are the proportionality parameters $\beta _{1}$ and $\beta _{2}$, the rate at which pathogenic bacteria are destroyed by autophagy and AMPs from epithelial cells $\beta _{3}$, the rate of uptake of macronutrients by commensal bacteria $\eta _{1}$, the rate of consumption of micronutrients by commensal bacteria $\eta _{3}$, the rate of uptake of micronutrients by pathogenic bacteria $\eta _{4}$, the rate of consumption of alternate nutrients by pathogens $\eta _{9}$ and the natural degradation rate of pro-inflammatory cytokines $\alpha _{11}$. An increase in $\beta _{1}$ and $\beta _{3}$ results in a decrease in the population of pathogenic bacteria (along with $\eta _{3}$), an increase in healthy epithelial cells (along with to $\eta _{3}$ and $\alpha _{11}$) and a decrease in pro-inflammatory cytokines (along with $\alpha _{11}$). An increase in $\beta _{2}$ and $\eta _{9}$ results in an increase in pathogens (along with $\eta _{1}$ and $\eta _{4}$), a decrease in healthy epithelial cells (along with $\eta _{1}$ and $\eta _{4}$) and an increase in pro-inflammatory cytokines (along with $\eta _{8}$).

### Supplementation

#### Vitamin D supplementation

Older adults are frequently cited as an at-risk population for vitamin D insufficiency and deficiency, and vitamin D supplementation in this age group is thought to improve innate and cell-mediated immunity. The impact of vitamin D supplementation *in silico* on individuals between the age of 20 and 80 years using the serum 25(OH)D concentrations predicted in Fig. 9a as the initial 25(OH)D level before supplementation at time $t=0$ days is therefore explored. Supplementation is implemented by increasing $D^{1}$ from its baseline value by a fixed amount, dependent upon dose, at $t=0$ up until $t=200$ days. Simulations showing the effect of the same dose and an age-related dose of vitamin D, given to individuals daily for 200 days, on the resulting serum levels of 25(OH)D is shown in Fig. 11. It is assumed that the dose is the same for both the UK and World threshold definitions.

A constant daily dose administered to all ages results in an increase in serum concentrations of 25(OH)D (solid lines in Fig. 11a and b) with individuals between 20 and 40 years attaining a healthy level after around 20 days (World) and between 20 and 60 years after approximately 25 days (UK). However, older adults (approximately over 60 (World) and 70 (UK) years) still have insufficient 25(OH)D levels in both cases after this time supporting the US National Academy of Medicine’s recommendation that higher daily allowances of vitamin D are required for older individuals to achieve adequate serum 25(OH)D levels^58^. Also note that all UK individuals do not attain serum concentrations of 75 nmol/L, thus failing to meet the World definition of healthy levels. The dashed lines in Fig. 11a and b show the same aged individuals but with older people receiving a higher dose supplement than younger ones. In this scenario, all individuals reach the same serum 25(OH)D levels (World) but older adults take longer to do so. An 80 year old would need to take a daily dose of almost twice as much as a 40 year old and over three times as much as a 20 year old to attain the same serum levels. The same doses administered to UK individuals results in higher serum 25(OH)D levels with increasing age so that an 80 year old has higher levels than a 20 year old, with individuals over 60 years attaining levels greater than 75 nmol/L.

Figures 12, 13 and 14 show the nutrient concentrations (Fig. 12a–h), bacterial populations (Fig. 12i–l), concentration of vitamin D and its metabolites (Fig. 13a-j), volume fractions of epithelial cells (Fig 14a-d) and immune response (Fig 14e-p) for the two dosing scenarios presented in Fig 11. With a constant daily dose of vitamin D supplements, concentrations of 25(OH)D and its metabolites in older adults increase, but still remain at insufficient levels. However, supplementation shows an improvement in inflammatory status in all ages, in particular in older adults over 60 years. An increase in 25(OH)D upregulates the VDR complex (Fig 13i and j) that helps repair the epithelial barrier via an increase in healthy epithelial cells and a corresponding decrease in damaged ones, as shown in Fig 14a-d. An increase in VDR also promotes the development of regulatory cells (Fig 14g and h) that help dampen down the immune response by increasing the production of anti-inflammatory cytokines. Additionally, upregulation in VDR inhibits T-cell proliferation (Fig 14i and j) and the pro-inflammatory cytokine production (Fig 14o and p), the latter impairing the activation of macrophages (Fig 14e and f) and B cells (Fig 14k and l). Supplementation does not significantly affect the concentration of macronutrients (Fig 12a and b) and micronutrients (Fig 12c and d), metabolites (Fig 12e and f) and alternate nutrients (Fig 12g and h) for younger adults but the latter two decrease in older age, resulting in a decrease in pathogenic bacteria (Fig 12k and l).

Administration of an age-related dose of vitamin D increases the concentration of the VDR complex, so that an 80 year old has similar levels to that of a 60 year old on a fixed dose. However, despite younger and older adults having similar serum concentrations of 25(OH)D with an age-related dose, the concentration of the VDR complex is much lower in older adults than in a younger individual. Higher VDR levels enhance the anti-inflammatory effect, improve the gut barrier integrity and reduce the population of pathogenic bacteria, resulting in a more effective resolution of inflammation.

Figure 15 shows the concentration of 25(OH)D, the healthy volume fraction of epithelial cells and the concentration of pro-inflammatory cytokines following administration of an age-related dose of vitamin D for 152 days (corresponding to current government UK advice to take supplements from October to early March) followed by a period of no supplements for the remainder of the year. As observed in Figs. 12, 13 and 14, there is an increase in the concentration of 25(OH)D and healthy epithelial cells and a decrease in the concentration of pro-inflammatory mediators during supplementation. However, following the cessation of vitamin D supplements, the system returns to its initial steady state.

#### Probiotic supplementation

Probiotics promote gut health via stimulation of epithelial innate immunity, and ageing does not appear to affect the ability of probiotics to elicit this protective response^41^. Administration of a constant daily dose of $\mathrm {5\times 10}^{9}$ CFU to all individuals independent of age is therefore included in the model. Supplementation is implemented at each age by increasing $P_{b}$ from zero at $t=0$ up until $t=200$ days, i.e. $P_{b}$ is represented by a Heaviside function

2 $$\begin{aligned} P_{b} = \left\{ \begin{array}{ll} \mathrm {5\times 10}^{9} \quad 0 \le t \le 200 \\ 0 \qquad \quad \ \ t < 0 \end{array} \right. \end{aligned}$$

It was observed in^36^ that higher CFU counts of probiotics do not necessarily improve health benefits over lower doses, and increases in the serum concentration of 25(OH)D and its metabolites, volume fraction of healthy epithelial cells and decrease in immune cell densities are not linear in the probiotic intake.

The effect of a daily administration of probiotics of dose $\mathrm {5\times 10}^{9}$ CFU/day for 200 days on the model variables is presented in Figs. 16, 17 and 18. As observed previously, predictions using the UK threshold definition for healthy serum 25(OH)D levels results in lower VDR:1,25$\text {(OH)}_{2}$D concentrations (Fig. 17e) and higher levels of inflammation (Fig. 18c-h) and intestinal epithelial barrier damage (Fig. 18b) than when using the World threshold definition. However, in both threshold scenarios, probiotic supplementation results in an increase in the VDR-complex concentration for all ages, with the largest increase observed in younger individuals. This dampens down inflammation, especially for older adults in whom a decrease in pro-inflammatory cytokines is observed (Fig. 18h) and corresponds to an increase in the volume fraction of healthy epithelial cells (Fig. 18a) and a decrease in immune cell densities (Fig. 18c,e-f) but to a lesser extent than that from vitamin D supplementation. There is an increase in commensals and a decrease in pathogen population, although too small to be observed in Fig. 16e and f. Probiotic supplementation increases the serum levels of 25(OH)D to healthy levels (both threshold definitions) for individuals aged under 50 years, with those over 50, still experiencing insufficient levels.

Simulation of a similar dosing regime (not shown) to that presented for vitamin D supplementation results in an analogous improvement during probiotic intake, followed by a return to the initial steady state values.

#### Combined vitamin D and probiotic supplementation

Simulations predicting the effect of combined vitamin D and probiotic supplements and comparing levels with those predicted with no supplements, vitamin D only and probiotics only are shown in Figs. 19, 20 and 21. Daily supplements are administered in combination on day 0 until day 200 under the following dosing scenarios and the response of the nutrient concentrations, bacteria populations, levels of vitamin D and its metabolites, the volume fraction of epithelial cells and the immune response following the intervention are predicted numerically:

- N—no supplements
- CVD—a constant daily vitamin D dose corresponding to an increase in production of 25(OH)D of 1.3 nmol/L day^−1^,
- AVD—an age-dependent vitamin D dose corresponding to an increase in production of 25(OH)D of 1.3 (20 years), 2 (40 years), 2.7 (60 years) and 3.7 (80 years) nmol/L day^−1^,
- P—a constant daily probiotics dose of $\mathrm {5\times 10}^{9}$ CFU/day,
- CCS—a constant daily dose corresponding to an increase in production of 25(OH)D of 1.3 nmol/L day^−1^ vitamin D and $\mathrm {5\times 10}^{9}$ CFU/day probiotics,
- ACS—an age-dependent dose corresponding to an increase in production of 25(OH)D of 1.3 (20 years), 2 (40 years), 2.7 (60 years) and 3.7 (80 years) nmol/L day^−1^ vitamin D and $\mathrm {5\times 10}^{9}$ CFU/day probiotics.

As with the individual supplementation described in the previous two subsections, co-supplementation upregulates the vitamin D receptor for all ages (Fig. 20), which helps repair the epithelial barrier function and stimulates the production of regulatory cells and anti-inflammatory cytokines (Fig. 21). An increase in VDR also inhibits T-cell proliferation and pro-inflammatory cytokine production, reduces the population of pathogenic bacteria (Fig. 19) and impairs the activation of macrophages and B cells (Fig. 21) with age-dependent dosing of vitamin D resulting in the best outcome.

There is little change in nutrient concentrations between dosing scenarios for each age group (Fig. 19). However, in older adults, the concentration of metabolites decreases with intervention due to there being an increased volume fraction of healthy epithelial cells utilising the SCFAs for proliferation. There is also a decrease in alternate nutrients as less metabolites are available for conversion by pathogens and the largest decrease occurs with age-dependent co-supplementation. However, there is only a small corresponding decrease in the pathogen population which cannot be observed with the colour scale used in Fig. 19. Nevertheless, a clear increase in pathogen numbers with age for all dosing regimens is observed.

The highest concentrations of serum 25(OH)D and VDR complex occur in young adults with combined probiotic and vitamin D supplementation and in the older population, with combined probiotic and age-dependent vitamin D supplementation (Fig. 20). The combination of vitamin D and probiotics results in healthy levels of serum 25(OH)D for older individuals for both World and UK threshold definitions, even when given the same dose of vitamin D as a younger individual. In the UK, an 80 year old also attains a serum 25(OH)D concentration of >75 nmol/L when given combined probiotic and age-dependent vitamin D supplementation, thereby meeting the World definition for healthy levels. In all dosing scenarios, the concentration of 25(OH)D and its metabolites is greatest in the young. This is also the case for the volume fraction of healthy epithelial cells where supplementation has no effect on the integrity of the epithelial barrier in younger individuals as it is undamaged. However, in older adults where the intestinal barrier has been compromised, supplementation has a positive impact on its repair with the most benefit being derived from age-dependent co-supplementation (Fig. 21).

As with the individual supplementation described in the previous two subsections, co-supplementation upregulates the vitamin D receptor for all ages which helps repair the epithelial barrier function and stimulates the production of regulatory cells and anti-inflammatory cytokines (Fig. 21). The pro-inflammatory cytokine concentration is highest in older individuals but decreases with co-supplementation. Anti-inflammatory mediators increase across all ages with supplementation and are highest in young adults taking combined probiotics and vitamin D. An increase in VDR also inhibits T-cell proliferation and pro-inflammatory cytokine production, reduces the population of pathogenic bacteria and impairs the activation of macrophages and B cells with age-dependent dosing of vitamin D resulting in the best outcome.

In summary, the model suggests that vitamin D supplements enhance the positive effects more than probiotics but taking them in combination results in the greatest benefit. There is also an increased advantage from taking these supplements in older age. Co-supplementation has synergistic effects on the concentrations of 25(OH)D and of its metabolites, on the density of regulatory cells and the concentration of anti-inflammatory cytokines, with the change in these model outputs being greater when vitamin D and probiotics are combined than the sum of the changes when administered separately. However, co-supplementation produces a combined effect that is less than the sum of the two separate supplements given individually for the remaining model outputs, most likely due to the lack of sensitivity to parameters preceding *R*, $V_{D_{a}}$ and *G* in the model equations (as shown by the local sensitivity analysis performed in Franks et al.^36^).

**Fig. 6:**
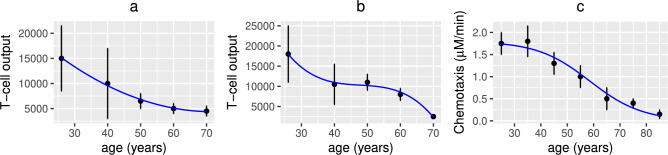
Measured rates of T-cell output and neutrophil chemotaxis with age. Fitted blue lines to experimental observations (approximated from Fig. 2 in^38^ and Fig 3A in^37^) for the T-cell output (men (**a**), women (**b**)) (sjTREC per million whole blood cells) and neutrophil chemotaxis (**c**) with age.

## Discussion

Poor health in an increasingly ageing population puts a huge burden on social and healthcare systems, along with increased morbidity and reduced quality of life. The principal aim of this study was to examine the age-related biological changes to the intestinal microbiota and immune system and assess the effect of simple nutritional interventions, i.e. probiotics and vitamin D supplementation, on the microbiota, vitamin D levels, immunosenencence and inflammageing.

Where available, clinical observations from the literature were used to develop age-dependent functional forms to describe how rate parameters alter with age. However, assumptions have been made for some parameters and a better understanding of how these change with age is essential for more quantitative predictions.

**Table 3:** Definition, baseline values and units for the epithelial barrier and immune response model parameters given in^36^.

Parameter	Description	Value & Units
$\epsilon _{1}$	Proliferation rate of intestinal epithelial cells	$\mathrm {4.9\times 10}^{-13}$ (ng day)^−1^
$\epsilon _{2}$	Rate of repair of damaged epithelial cells by VDR	$\mathrm {2\times 10}^{9}$ ml/(ng day)
$\epsilon _{3}$	Removal rate of damaged epithelial cells by macrophages	$\mathrm {3.17\times 10}^{-6}$ ml/day
$\epsilon _{4}$	Damage to epithelial cells by pro-inflammatory mediators	$\mathrm {2.7\times 10}^{3}$ ml/(ng day)
$\epsilon _{5}$	Damage to epithelial cells by pathogenic bacteria	$\mathrm {1\times 10}^{-12}$ (CFU day)^−1^
$\iota _{1}$	Rate of activation of macrophages by pro-inflammatory cytokines	$\mathrm {1.35\times 10}^{9}$ (ng day)^−1^
$\iota _{2}$	Rate of inhibition of macrophages by anti-inflammatory cytokines	$\mathrm {1.13\times 10}^{2}$ ml/(ng day)
$\iota _{3}$	Natural death rate of macrophages	1 day^−1^
$\iota _{4}$	Rate that T/B cells are differentiated into T/B regulatory cells in presence of VDR	$\mathrm {2.38\times 10}^{11}$ (ng day)^−1^
$\iota _{5}$	Rate that T/B cells are differentiated into T/B regulatory cells in presence of commensal bacteria	$\mathrm {8.5\times 10}^{-11}$ (CFU ml day)^−1^
$\iota _{6}$	Rate at which differentiation into regulatory cells is inhibited by pathogenic bacteria	$\mathrm {1\times 10}^{-14}$ CFU^−1^
$\iota _{7}$	Combined natural death rate of T/B regulatory cells	1 day^−1^
$\iota _{8}$	Rate of utilisation of metabolites for T-helper cell proliferation	$\mathrm {2.57\times 10}^{-8}$ (ng ml day)^−1^
$\iota _{9}$	Rate of T-helper cell proliferation in response to pro-inflammatory cytokines	$\mathrm {1.12\times 10}^{9}$ (ng day)^−1^
$\iota _{10}$	Rate of T-helper cell proliferation in response to pathogenic bacteria	$\mathrm {1.05\times 10}^{-7}$ (CFU ml day)^−1^
$\iota _{11}$	Rate of inhibition to T helper cell proliferation by VDR	$\mathrm {4\times 10}^{6}$ ml/ng
$\iota _{12}$	Rate of inhibition to T helper cell proliferation by commensals	$\mathrm {1\times 10}^{-15}$ CFU^−1^
$\iota _{13}$	Natural rate of T-helper cell death	10 day^−1^
$\iota _{14}$	Rate of activation of plasma B cells by bacterial products	$\mathrm {1.28\times 10}^{-8}$ (ng ml day)^−1^
$\iota _{15}$	Rate of maturation of plasma B cells in presence of pro-inflammatory cytokines	$\mathrm {5\times 10}^{8}$ (ng day)^−1^
$\iota _{16}$	Rate of inhibition of plasma B cells by VDR	$\mathrm {4\times 10}^{8}$ ml/ng
$\iota _{17}$	Natural death rate of plasma B cells	0.81 day^−1^
$\alpha _{1}$	Production rate of anti-inflammatory cytokines by epithelial cells upregulated by VDR	$\mathrm {7.08\times 10}^{5}$ day^−1^
$\alpha _{2}$	Production rate of anti-inflammatory cytokines by T and B regulatory cells stimulated by commensal bacteria	$\mathrm {2.07\times 10}^{-21}$ ng/(CFU day)
$\alpha _{3}$	Production rate of anti-inflammatory cytokines by macrophages after consuming damaged epithelial cells	$\mathrm {1.13\times 10}^{-7}$ ng/day
$\alpha _{4}$	Production rate of anti-inflammatory cytokines by macrophages after consuming pathogenic bacteria	$\mathrm {1.69\times 10}^{-20}$ ng/(CFU day)
$\alpha _{5}$	Natural degradation rate of anti-inflammatory cytokines	$\mathrm {7.5\times 10}^{2}$ day^−1^
$\alpha _{6}$	Production rate of pro-inflammatory cytokines in response to damaged epithelial cells	$\mathrm {9.3\times 10}^{-18}$ ng/(CFU ml day)
$\alpha _{7}$	Production rate of pro-inflammatory cytokines by activated innate immune cells	$\mathrm {5.94\times 10}^{-11}$ ng/day
$\alpha _{8}$	Inhibition of pro-inflammatory cytokines by VDR	$\mathrm {4\times 10}^{7}$ ml/ng
$\alpha _{9}$	Inhibition of pro-inflammatory cytokines by commensals	$\mathrm {5\times 10}^{-15}$ CFU^−1^
$\alpha _{10}$	Production rate of pro-inflammatory cytokines by T helper cells	$\mathrm {8.78\times 10}^{-24}$ ng/(CFU day)
$\alpha _{11}$	Natural degradation rate of pro-inflammatory cytokines	1.2 day^−1^
$\alpha _{c}$	Rate of pro-inflammatory cytokine release by damaged epithelial cells	$\mathrm {2.3\times 10}^{-3}$ ng/(ml day)
$\alpha _{g}$	Rate of anti-inflammatory cytokine release by healthy epithelial cells	$\mathrm {1.77\times 10}^{-3}$ ng/(ml day)
$\gamma _{c}$	Background production rate of pro-inflammatory cytokines	$\mathrm {3\times 10}^{-4}$ ng/(ml day)
$\gamma _{g}$	Background production rate of anti-inflammatory cytokines	0.35 ng/(ml day)

**Table 4:** Comparison of key age-related model outputs with results in the literature.

Model output	Literature source
Loss of intestinal barrier function	^41,71,72^
Increase in pro-inflammatory bacteria	^70^
Decrease in serum 25(OH)D levels	^53^
Increase in pro-inflammatory cytokines (e.g. IL-6, IL-1, TNF-$\alpha$, C-RP)	^69^
Functional decline in innate immune system (i.e. macrophages)	^1–3^
Functional decline in adaptive immune system (i.e. B- and T-cells)	^1–3^

Nevertheless, the model can predict differences in the intestinal microbiota, vitamin D status and immune response across adulthood. It shows the age-related functional decline of the innate and adaptive immune system, loss of intestinal barrier function and elevation in systemic inflammation with ageing, predicting that the older population have more opportunistic pro-inflammatory bacteria and higher levels of pro-inflammatory mediators than younger individuals. This can result in the translocation of pathogenic bacteria and their structural components into the bloodstream causing inflammation throughout the body. The structural complexity and functional capability of the intestinal microbiota decline with age and is likely a factor causing immunosenescence. However, this study has highlighted the need for further clinical studies examining the relationship between age and changes in the microbiota, gastrointestinal tract function and immune response.

Vitamin D levels are generally low amongst adults, in particular older people due to the reduced generation of vitamin D in the skin, decline in renal function and reduction of binding of VDR to 1,25(OH)_2_D. The model has been able to assess the health impact of the lower threshold definition used in the UK for healthy serum 25(OH)D levels compared to elsewhere, and has predicted an increase in epithelial barrier damage and higher inflammation increasing the likelihood of ill health caused by inflammageing. Our model predicts a gradual decline in serum vitamin D levels with age, resulting in individuals aged over 60 years becoming vitamin D deficient. However, it also implies that administration of vitamin D supplements has significant benefits to older adults (and less of an effect on young healthy individuals), with age-related doses helping to attain the desired healthy serum levels: the supplement-related increase in serum 25(OH)D upregulates the VDR complex, which enhances barrier function and improves innate and cell-mediated immunity helping to prevent low-grade inflammation. In modelling the dose of vitamin D as a step function, our model suggests that seasonal administration of vitamin D supplements is enough to increase levels of 25(OH)D, but individuals who have limited exposure to sunlight during the spring and summer months should continue to take supplements throughout the year to avoid becoming deficient. Vitamin D is stored in adipose tissue and the liver until required, so a more biologically plausible scenario is for there to be a gradual decline in available vitamin D following cessation of supplements. Similarly, the effective benefit on the immune system, metabolism and microbiota will take time e.g. for the absorption and tissue distribution of 25(OH)D, regulation of genes, changes to bacterial populations etc, so future consideration of a gradual decline in dose and delayed onset compartments would be an interesting extension to the model.

Our model also suggests that administration of specific probiotics positively affects the immune system by supporting the maintenance of immune cells and intestinal barrier function and protecting against intestinal inflammation by mediating inflammatory signalling molecules. Moreover, it indicates that co-supplementation of vitamin D and probiotics increases the positive effects for all ages but is more advantageous in the older population. However, the combined effect of co-supplementation was found to be less than the sum of the two separate supplements administered individually, suggesting some overlapping effects.

The relationship between the intestinal microbiota and human health during ageing is an area of increasing interest and to the best of our knowledge, our model is the first to explore the complex interactions between the various mechanistic components with age and to determine the impact of manipulating the intestinal microbiota with dietary components. Despite a lack of data in this area, the model produces biologically realistic predictions and suggests how simple nutritional interventions can potentially be of therapeutic benefit to older people. However, changes to the microbiome during ageing involve nuanced community transitions, for example loss of SCFA producers, rise in facultative anaerobes etc, so considering a broader range of functionally grouped or taxonomically resolved populations (instead of the commensals and pathogens considered in this paper) would better capture the microbial ecology and diet-microbe-host interactions.

The model explores hypothetical supplementation scenarios for vitamin D and probiotics but could be adapted to simulate other public health interventions, for example dietary fibre intake (which increases the concentration of SCFAs i.e. Nmb in our model) and physical activity (which increases microbial diversity, promotes bacteria that produce short-chain fatty acids (SCFAs), enhances metabolic function i.e. individuals are better at fermenting dietary fibre, reduces pro-inflammatory bacteria and enhances populations associated with anti-inflammatory pathways—note that this is dependent upon exercise intensity). The combination of exercise with a fibre-rich diet to maximize beneficial microbial changes would be an interesting area of future study.

**Fig. 7:**
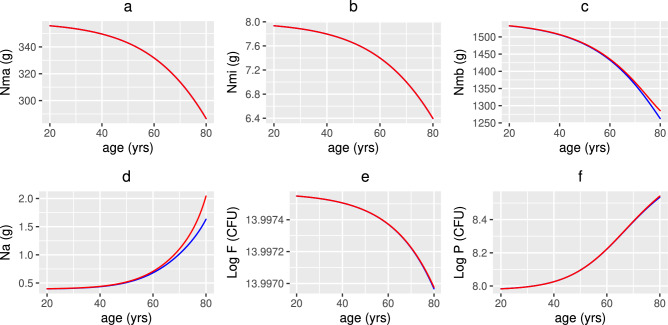
Numerical simulations of nutrients and bacteria with age. The predicted concentrations of (**a**) macronutrients, (**b**) micronutrients, (**c**) metabolites, (**d**) alternate nutrients and populations of (**e**) commensals and (**f**) pathogens with age, from solving Eqs. (3)–(23) with baseline parameters given in Tables 1, 2 and 3 and age functions given in Figs. 2, 3, 4 and 5. The production rates $D^{0}$ and $D^{1}$ to attain a concentration of 50 nmol/L (red line) and 75 nmol/L (blue line) 25(OH)D at age 20 years are $D^{0}=3.2$, 4.8 and $D^{1}=0.35$, 0.5 nmol/L day^−1^, respectively. Note that 1 nmol/L of 25(OH)D = 2.5 ng/ml.

**Fig. 8:**
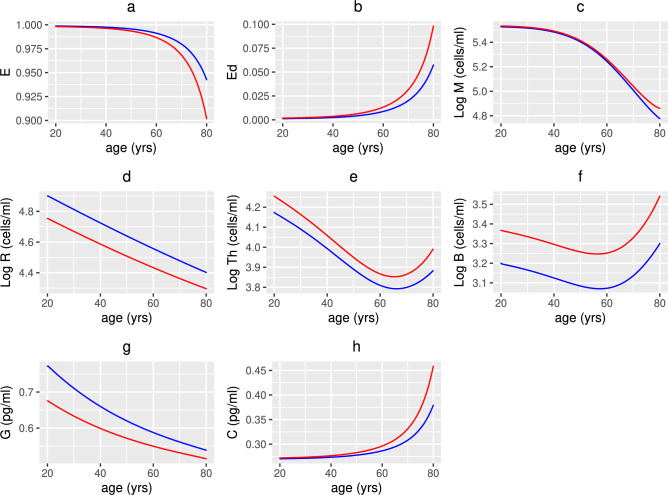
Numerical simulations of epithelial cells, densities of immune cells and concentrations of cytokines with age. The predicted volume fractions of (**a**) healthy and (**b**) damaged epithelial cells, densities of (**c**) macrophages, (**d**) regulatory T-cells, (**e**) T-cells and (**f**) plasma B-cells and concentrations of (**g**) anti- and (**h**) pro-inflammatory cytokines with age, from solving Eqs. (3)–(23) with baseline parameters given in Tables 1, 2 and 3 and age functions given in Figs. 2, 3, 4 and 5. The production rates $D^{0}$ and $D^{1}$ to attain a concentration of 50 nmol/L (red line) and 75 nmol/L (blue line) 25(OH)D at age 20 years are $D^{0}=3.2$, 4.8 and $D^{1}=0.35$, 0.5 nmol/L day^−1^, respectively.

**Fig. 9:**
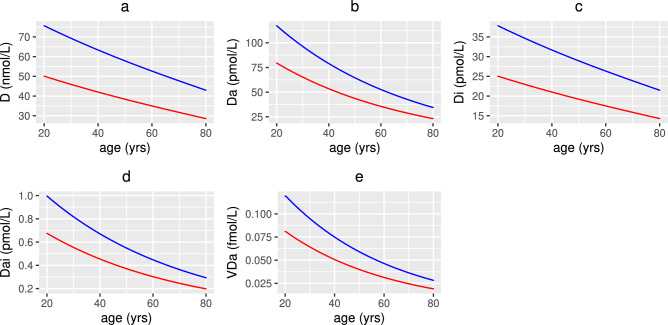
Numerical simulations of 25(OH)D and its metabolites with age. The predicted concentrations of (**a**) extracellular 25(OH)D, (**b**) extracellular 1,25(OH)_2_D, (**c**) intracellular 25(OH)D, (**d**) intracellular 1,25(OH)_2_D and (**e**) the VDR:1,25(OH)_2_D complex with age, from solving Eqs. (3)–(23) with baseline parameters given in Tables 1, 2 and 3 and age functions given in Figs. 2, 3, 4 and 5. The production rates $D^{0}$ and $D^{1}$ to attain a concentration of 50 nmol/L (red line) and 75 nmol/L (blue line) 25(OH)D at age 20 years are $D^{0}=3.2$, 4.8 and $D^{1}=0.35$, 0.5 nmol/L day^−1^, respectively.

**Fig. 10:**
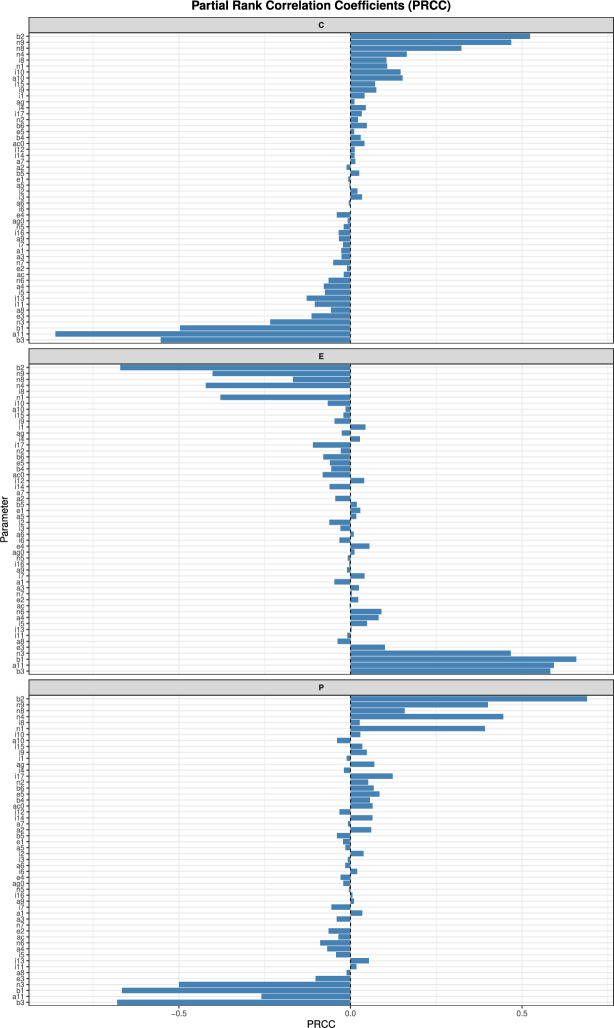
Influence of model parameters on key outputs Partial Rank Correlation Coefficients (PRCCs), showing the influence of model parameters on key outputs. Bars indicate the strength and direction of the monotonic relationship between each parameter and the final value of the population of pathogenic bacteria (P), volume fraction of healthy epithelial cells (E) and concentration of pro-inflammatory cytokines (C). Model Eqs. (3)–(23) were solved at age 60 years. Larger absolute values correspond to greater influence.

## Methods

One of the key consequences of ageing is a decline in immune function. The effects on the immune system are widespread and older individuals (those aged over 65) often do not respond efficiently to new or previously encountered antigens. There are several components of ageing which relate to the immune system^42^.

- Immunosenescence, which is a change in the quality and quantity of the immune response, results from an imbalance in the number and type of immune cells, their ability to mount an adequate response against pathogens (or other sources of antigens such as vaccines) and to retain immune memory of previous pathogens encountered. It is also characterised by a chronic inflammatory response and ineffective resolution of inflammation, favouring a pro-inflammatory state, which is referred to as inflammageing.
- Inflammageing is driven by three aspects of immunosenescence: a dysregulation of the innate monocyte-macrophage network resulting in a disposition towards inflammatory responses, a gradual senescence of T- and B-cells, and external amplifying factors such as the chronic exposure to antigens and inflammatory stimuli.
- The immune system is the orchestrator of the collaboration between gut microbiota and its host, also acting as a surveillance system to ensure microbial tolerance and absent or attenuated responses to commensal bacteria while mounting vigorous and sterilising responses to pathogens. There is a dynamic and bi-directional interaction, with alterations in commensal gut bacteria (dysbiosis) impacting on immune regulation and function. Age-affected microbiota can promote inflammation^43^ and reversing these age-related microbiota changes represents a potential strategy for reducing age-associated inflammation associated morbidity. Microbial dysbiosis affects the production of anti-inflammatory cytokines, vitamins, and immune cells, exacerbating low-grade inflammation and the ageing process in the gut. This reduces gut barrier integrity and increases susceptibility to infections.

The model in this section builds upon the model presented in^36^, which captures the interactions between the microbiome, vitamin D receptor (VDR) and the immune response. Fig. S1 summarises the complex interactions between these three components. The relevant modelling is summarised below, presenting the possible effects of ageing on each of its components.

**Fig. 11:**
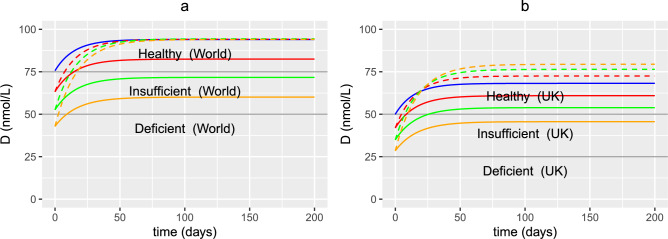
Predicted effects of vitamin D supplementation on 25(OH)D with age. Simulations predicting the effect of the same daily dose (solid lines) and an age-dependent daily dose (dashed lines) of vitamin D supplements given over 200 days on individuals with age-dependent initial serum concentrations of 25(OH)D determined from Fig. 9a for (**a**) World and (**b**) UK threshold definitions. The blue, red, green and orange lines represent 20, 40, 60 and 80 years old, respectively. Baseline parameters are given in Tables 1, 2 and 3 and baseline values (no supplementation) for vitamin D intake in (**a**) are $D^{0}=4.8$ and $D^{1}=0.5$ nmol/L day^−1^ and in (**b**), $D^{0}=3.2$ and $D^{1}=0.35$ nmol/L day^−1^. The fixed increase in production of 25(OH)D from supplements (i.e. the increase in $D^{1}$) is 1.3 nmol/L day^−1^ (solid lines) and the age dependent increase is 1.3 nmol/L day^−1^ at 20 years, 2 nmol/L day^−1^ at 40 years, 2.7 nmol/L day^−1^ at 60 years and 3.7 nmol/L day^−1^ at 80 years (dashed lines).

**Fig. 12:**
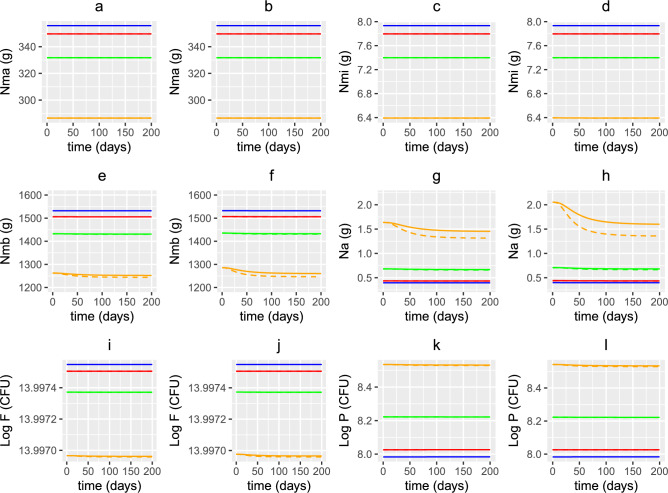
Predicted effects of vitamin D supplementation on nutrients and bacteria with age. The predicted concentrations of (**a**, **b**) macronutrients, (**c**, **d**) micronutrients, (**e**, **f**) metabolites, (**g**, **h**) alternate nutrients and populations of (**i**, **j**) commensals and (**k**, **l**) pathogens with age, from solving Eqs. (3)–(23) with baseline parameters given in Tables 1, 2 and 3 and age functions given in Figs. 2, 3, 4 and 5. A constant (solid lines) or age-dependent (dashed lines) daily intervention of vitamin D supplements is administered from day 0 to day 200 to individuals aged 20 (blue), 40 (red), 60 (green) and 80 (orange) years old. Baseline values (no supplementation) for vitamin D intake in (**a**, **c**, **e**, **g**, **i**, **k**) are $D^{0}=4.8$ and $D^{1}=0.5$ nmol/L day^−1^ and in (**b**, **d**, **f**, **h**, **j**, **l**) are $D^{0}=3.2$ and $D^{1}=0.35$ nmol/L day^−1^. The fixed increase in production of 25(OH)D from supplements across all ages is 1.3 nmol/L day^−1^ (solid lines) and the age dependent increase is 1.3 nmol/L day^−1^ at 20 years, 2 nmol/L day^−1^ at 40 years, 2.7 nmol/L day^−1^ at 60 years and 3.7 nmol/L day^−1^ at 80 years (dashed lines).

**Fig. 13:**
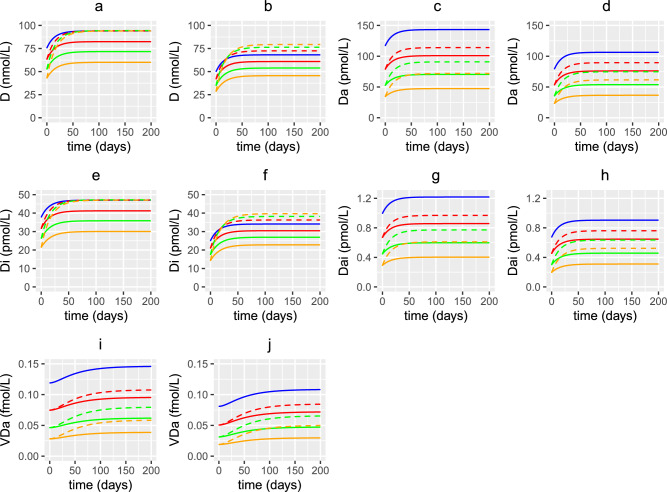
Predicted effects of vitamin D supplementation on 25(OH)D and its metabolites with age. The predicted concentrations of (**a**, **b**) extracellular 25(OH)D, (**c**, **d**) extracellular 1,25(OH)_2_D, (**e**, **f**) intracellular 25(OH)D, (**g**, **h**) intracellular 1,25(OH)_2_D and (**i**, **j**) the VDR:1,25(OH)_2_D complex with age, from solving Eqs. (3)–(23) with baseline parameters given in Tables 1, 2 and 3 and age functions given in Figs. 2, 3, 4 and 5. A constant (solid lines) or age-dependent (dashed lines) daily intervention of vitamin D supplements is administered from day 0 to day 200 to individuals aged 20 (blue), 40 (red), 60 (green) and 80 (orange) years old. Baseline values (no supplementation) for vitamin D intake in (a,c,e,g,i,k) are $D^{0}=4.8$ and $D^{1}=0.5$ nmol/L day^−1^ and in (b,d,f,h,j,l) are $D^{0}=3.2$ and $D^{1}=0.35$ nmol/L day^−1^. The fixed increase in production of 25(OH)D from supplements across all ages is 1.3 nmol/L day^−1^ (solid lines) and the age dependent increase is 1.3 nmol/L day^−1^ at 20 years, 2 nmol/L day^−1^ at 40 years, 2.7 nmol/L day^−1^ at 60 years and 3.7 nmol/L day^−1^ at 80 years (dashed lines).

**Fig. 14:**
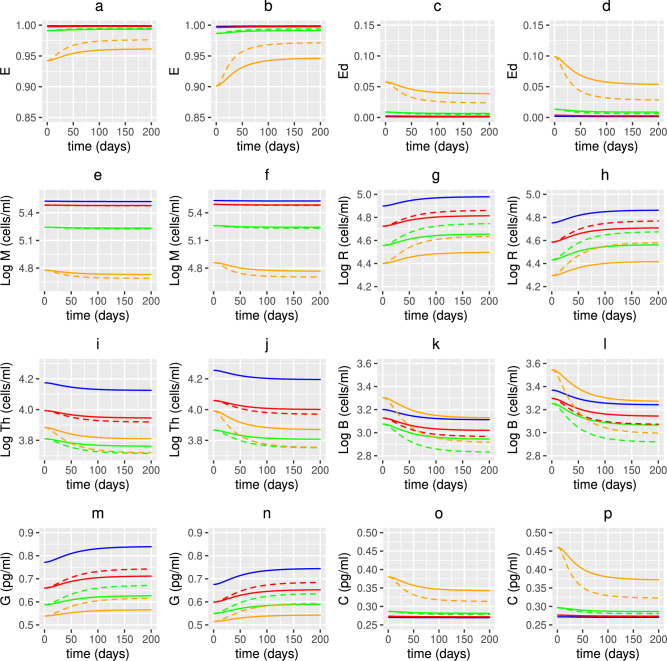
Predicted effects of vitamin D supplementation on epithelial cells, immune cell densities and cytokine concentrations with age. The predicted volume fraction of (**a**, **b**) healthy and (**c**, **d**) damaged epithelial cells, densities of (**e**, **f**) macrophages, (**g**, **h**) regulatory cells, (**i**, **j**) T-cells and (**k**, **l**) plasma B-cells and concentrations of (**m**, **n**) anti- and (**o**, **p**) pro-inflammatory cytokines with age, from solving Eqs. (3)–(23) with baseline parameters given in Tables 1, 2 and 3 and age functions given in Figs. 2, 3, 4 and 5. A constant (solid lines) or age-dependent (dashed lines) daily intervention of vitamin D supplements is administered from day 0 to day 200 to individuals aged 20 (blue), 40 (red), 60 (green) and 80 (orange) years old. Baseline values (no supplementation) for vitamin D intake in (**a**, **c**, **e**, **g**, **i**, **k**, **m**, **o**) are $D^{0}=4.8$ and $D^{1}=0.5$ nmol/L day^−1^ and in (**b**, **d**, **f**, **h**, **j**, **l**, **n**, **p**) are $D^{0}=3.2$ and $D^{1}=0.35$ nmol/L day^−1^. The fixed increase in production of 25(OH)D from supplements across all ages is 1.3 nmol/L day^−1^ (solid lines) and the age dependent increase is 1.3 nmol/L day^−1^ at 20 years, 2 nmol/L day^−1^ at 40 years, 2.7 nmol/L day^−1^ at 60 years and 3.7 nmol/L day^−1^ at 80 years (dashed lines).

### The microbiota

Older adults generally have lower caloric intake as they are less physically active, but increased essential nutrient and micronutrient needs compared to younger adults as they are less able to absorb or utilise them. This is due to a combination of age-related factors including changes in gastrointestinal tract physiology (e.g. changes in gastric emptying and changes in co-factor production—for example digestive enzymes, mucins, hormones, neurotransmitters etc. in the gut affect absorption) and resistance in metabolism (e.g. anabolic resistance in muscle means older people need more protein than younger adults). Nutrient needs in this population are also affected by chronic health conditions, use of multiple medicines, and changes in body composition. Intake of micronutrients from 20 to 60 years old remains constant^44^ but diet quality is generally reduced with ageing, and older adults are prone to insufficient energy, macronutrient (protein), and micronutrient (vitamin B12, iron, vitamin D and calcium) intakes, more significantly after the age of 65 years^42^. However, researchers have found that there is considerable variation in micro- and macro-nutrient intake between individuals at all ages^45^. Changes in diet and nutrient intake bring about rapid changes in the intestinal microbiome and, in extreme cases of nutrient depletion, intestinal bacteria can switch their substrata preference to host derived substrates, feeding off mucins and other host cell glycans^46^. This can result in deterioration of the intestinal barrier and “leaky gut” but this scenario is not considered here.

The gut microbiome is a major source of vitamins and particularly vitamin B12 and there is a competitive dynamic between the microbiome and its host, which likely changes with age, for these essential micronutrients in the gut. The small intestine is the major site of nutrient extraction and uptake that results in only resistant undigestible food substrates reaching the colon for fermentative breakdown.

In^36^, intestinal microbes and epithelial cells compete for micronutrients, taking them up at rates $\eta _{3}$ (commensals), $\eta _{4}$ (pathogens) and $\eta _{5}$ (epithelial cells). It is not known how this competition changes with age but might be a consequence of alterations to the microbiome composition (i.e. ratio between commensal (i.e. beneficial) (F) and pathogenic (or pro-inflammatory) (P) bacteria) and volume fraction of healthy epithelial cells (E). It is therefore assumed that these rate parameters are constant (i.e. not age dependent) and that F, P and E change with age. It is also assumed that the consumption rate of nutrients by commensal bacteria ($\eta _{1}$, $\eta _{3}$, $\eta _{7}$), by pathogens ($\eta _{4}$, $\eta _{9}$) and by epithelial cells ($\eta _{5}$), the rate of release of nutrients from degradation of pathogens ($\eta _{2}$) and the faecal removal rate of excess nutrients from the gastrointestinal tract (*q*) are independent of age so these parameters remain constant. There is however, an age-related decline in the absorption of nutrients, so a reduction in the rates of macronutrients $N_{ma}^{0}$ and micronutrients $N_{mi}^{0}$ entering into the gastrointestinal tract in older adults is assumed.

**Fig. 15:**
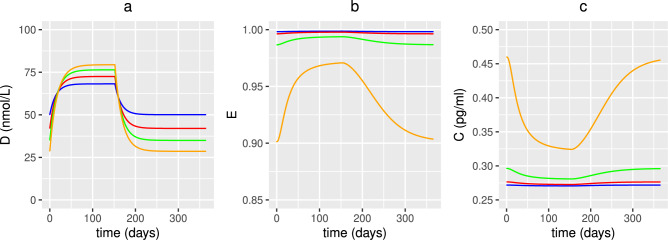
Predicted effects of vitamin D supplementation on 25(OH)D, healthy epithelial cells and pro-inflammatory cytokine concentration with age for seasonal dosing regime. The predicted concentration of extracellular 25(OH)D (**a**), volume fraction of healthy epithelial cells (**b**) and concentration of pro-inflammatory cytokines (**c**) with age, from solving Eqs. (3)–(23) with baseline parameters given in Tables 1, 2 and 3 and age functions given in Figs. 2, 3, 4 and 5. An age-dependent daily intervention of vitamin D supplements is administered from day 0 to day 152 to individuals aged 20 (blue), 40 (red), 60 (green) and 80 (orange) years old, followed by a period of no intervention from day 153 to day 365. Baseline values (no supplementation) for vitamin D intake in are $D^{0}=4.8$ and $D^{1}=0.5$ nmol/L day^−1^. The age-dependent increase in production of 25(OH)D from supplements across all ages from day 0-152 is 1.3 nmol/L day^−1^ at 20 years, 2 nmol/L day^−1^ at 40 years, 2.7 nmol/L day^−1^ at 60 years and 3.7 nmol/L day^−1^ at 80 years.

**Fig. 16:**
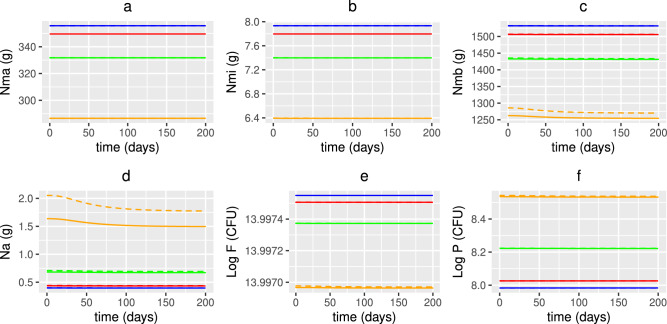
Predicted effects of probiotic supplementation on nutrients and bacteria with age. The predicted concentrations of (**a**) macronutrients, (**b**) micronutrients, (**c**) metabolites, (**d**) alternate nutrients and populations of (**e**) commensals and (**f**) pathogens with age, from solving Eqs. (3)–(23) with baseline parameters given in Tables 1, 2 and 3 and age functions given in Figs. 2, 3, 4 and 5. Solid lines represent baseline values of vitamin D intake $D^{0}=4.8$ and $D^{1}=0.5$ nmol/L day^−1^ and dashed lines $D^{0}=3.2$ and $D^{1}=0.35$ nmol/L day^−1^. A constant daily intervention of probiotics $\mathrm {5\times 10}^{9}$ CFU/day is administered from day 0 to day 200 to individuals aged 20 (blue), 40 (red), 60 (green) and 80 (orange) years.

**Fig. 17:**
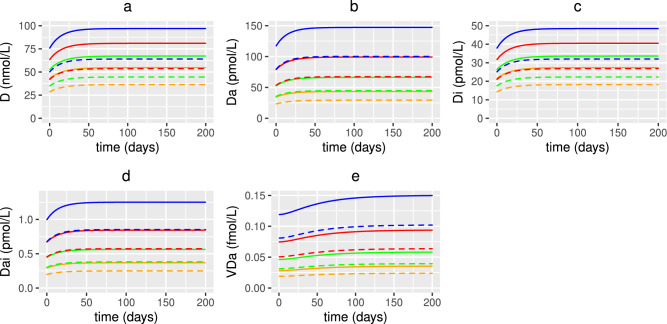
Predicted effects of probiotic supplementation on 25(OH)D and its metabolites with age. The predicted concentrations of (**a**) extracellular 25(OH)D, (**b**) extracellular 1,25(OH)_2_D, (**c**) intracellular 25(OH)D, (**d**) intracellular 1,25(OH)_2_D and (**e**) the VDR:1,25(OH)_2_D complex with age, from solving Eqs. (3)–(23) with baseline parameters given in Tables 1, 2 and 3 and age functions given in Figs. 2, 3, 4 and 5. Solid lines represent baseline values of vitamin D intake $D^{0}=4.8$ and $D^{1}=0.5$ nmol/L day^−1^ and dashed lines $D^{0}=3.2$ and $D^{1}=0.35$ nmol/L day^−1^. A constant daily intervention of probiotics $\mathrm {5\times 10}^{9}$ CFU/day is administered from day 0 to day 200 to individuals aged 20 (blue), 40 (red), 60 (green) and 80 (orange) years.

**Fig. 18:**
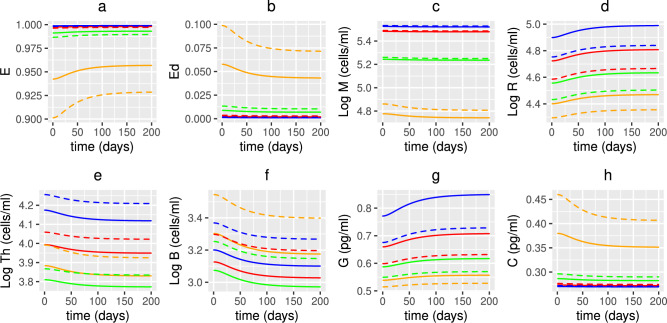
Predicted effects of probiotic supplementation on epithelial cells, immune cell densities and cytokine concentrations with age. The predicted volume fraction of (**a**) healthy and (**b**) damaged epithelial cells, densities of (**c**) macrophages, (**d**) regulatory cells, (**e**) T-cells and (**f**) plasma B-cells and concentrations of (**g**) anti- and (**h**) pro-inflammatory cytokines with age, from solving Eqs. (3)–(23) with baseline parameters given in Tables 1, 2 and 3 and age functions given in Figs. 2, 3, 4 and 5. Solid lines represent baseline values of vitamin D intake $D^{0}=4.8$ and $D^{1}=0.5$ nmol/L day^−1^ and dashed lines $D^{0}=3.2$ and $D^{1}=0.35$ nmol/L day^−1^. A constant daily intervention of probiotics $\mathrm {5\times 10}^{9}$ CFU/day is administered from day 0 to day 200 to individuals aged 20 (blue), 40 (red), 60 (green) and 80 (orange) years.

**Fig. 19:**
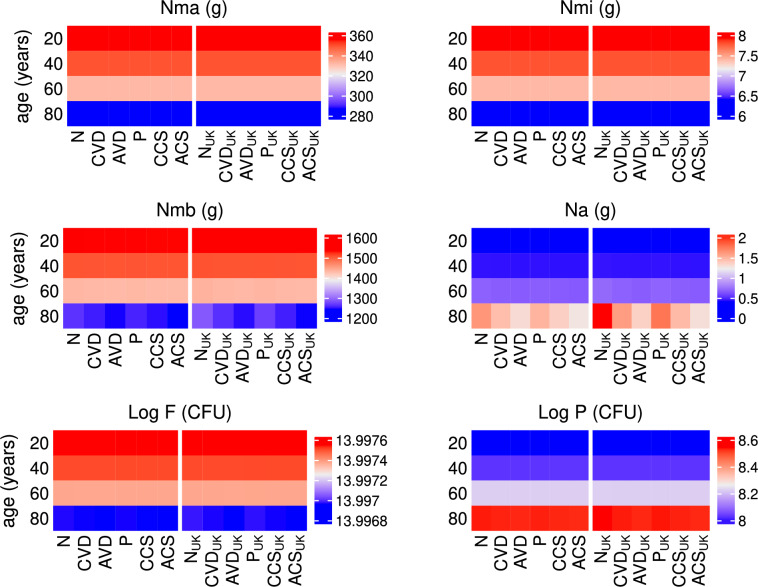
Predicted effects of co-supplementation on nutrients with age. The predicted steady state values on day 200 of the concentration of macronutrients ($N_{ma}$), micronutrients ($N_{mi}$), metabolites ($N_{mb}$) and alternate nutrients ($N_{a}$) from solving Eqs. (3)–(23) with baseline parameters given in Tables 1, 2 and 3 and age functions given in Figs. 2, 3, 4 and 5. A daily intervention is administered from day 0 to day 200 to individuals aged 20-80 years for dosing regimens N, CVD, AVD, P, CCS and ACS with baseline values of vitamin D intake $D^{0}=4.8$ and $D^{1}=0.5$ nmol/L day^−1^ (no subscript on horizontal axis labels) and $D^{0}=3.2$ and $D^{1}=0.35$ nmol/L day^−1^ (subscript _UK_ on horizontal axis labels).

**Fig. 20:**
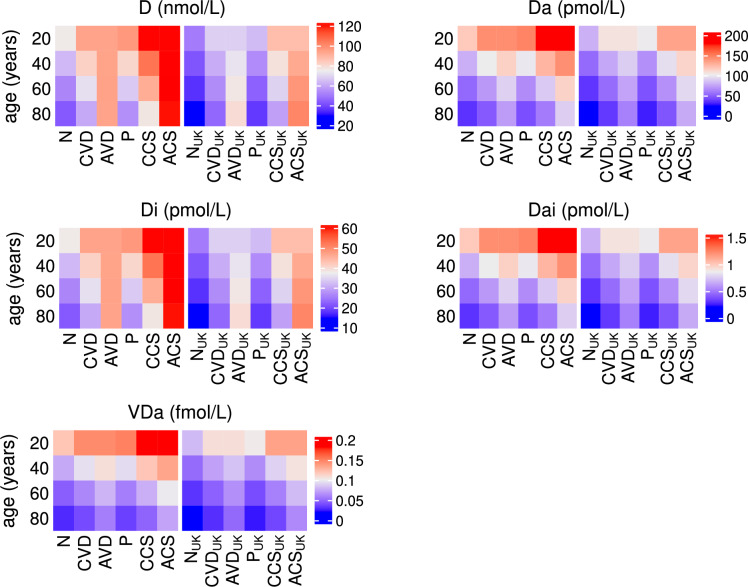
Predicted effects of co-supplementation on 25(OH)D and its metabolites with age. The predicted steady state values on day 200 of the concentration of extracellular 25(OH)D (D), extracellular 1,25(OH)_2_D ($D_{a}$), intracellular 25(OH)D ($D_{i}$), intracellular 1,25(OH)_2_D ($D_{a_{i}}$) and the VDR:1,25(OH)_2_D complex ($V_{D_{a}}$) with age, from solving Eqs. (3)–(23) with baseline parameters given in Tables 1, 2 and 3 and age functions given in Figs. 2, 3, 4 and 5. A daily intervention is administered from day 0 to day 200 to individuals aged 20-80 years for dosing regimens N, CVD, AVD, P, CCS and ACS with baseline values of vitamin D intake $D^{0}=4.8$ and $D^{1}=0.5$ nmol/L day^−1^ (no subscript on horizontal axis labels) and $D^{0}=3.2$ and $D^{1}=0.35$ nmol/L day^−1^ (subscript _UK_ on horizontal axis labels).

**Fig. 21:**
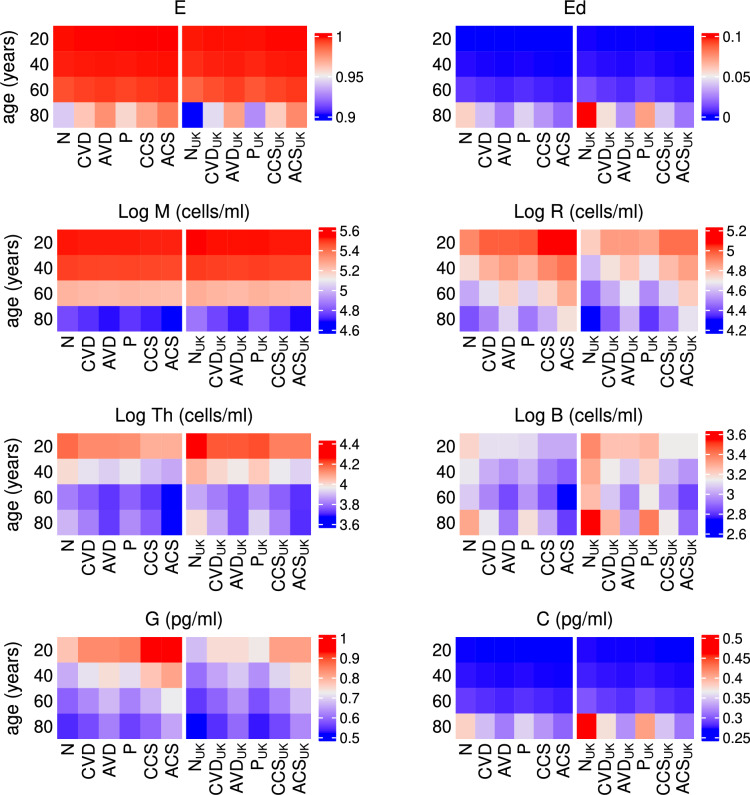
Predicted effects of co-supplementation on epithelial cells, immune cell densities and cytokine concentrations with age. The predicted steady state values on day 200 of the volume fraction of healthy (E) and damaged epithelial cells ($E_{d}$), densities of macrophages (M), regulatory cells (R), T-cells ($T_{h}$) and plasma B-cells (B) and concentrations of anti- (G) and pro-inflammatory cytokines (C) with age, from solving Eqs. (3)–(23) with baseline parameters given in Tables 1, 2 and 3 and age functions given in Figs. 2, 3, 4 and 5. A daily intervention is administered from day 0 to day 200 to individuals aged 20-80 years for dosing regimens N, CVD, AVD, P, CCS and ACS with baseline values of $D^{0}=4.8$ and $D^{1}=0.5$ nmol/L day^−1^ (no subscript on horizontal axis labels) and $D^{0}=3.2$ and $D^{1}=0.35$ nmol/L day^−1^ (subscript _UK_ on horizontal axis labels).

The equations governing the concentrations of macronutrients ($N_{ma}$), micronutrients ($N_{mi}$), metabolites ($N_{mb}$) and alternate nutrients ($N_{a}$) over an individual’s adult lifespan (i.e. age 20 to 80 years) are then

3 $$\begin{aligned} \frac{dN_{ma}}{dt}= & f_{1}(t)N_{ma}^{0}-\eta _{1}FN_{ma}-qN_{ma}, \end{aligned}$$

4 $$\begin{aligned} \frac{dN_{mi}}{dt}= & f_{1}(t)N_{mi}^{0}+\eta _{2}N_{mb}EP-\eta _{3}FN_{mi}-\eta _{4}PN_{mi}-\eta _{5}EN_{mi}-qN_{mi}, \end{aligned}$$

5 $$\begin{aligned} \frac{dN_{mb}}{dt}= & \eta _{1}FN_{ma}-\eta _{6}EN_{mb}-\eta _{7}FN_{mb}-\eta _{8}N_{mb}CP-qN_{mb}, \end{aligned}$$

6 $$\begin{aligned} \frac{dN_{a}}{dt}= & \eta _{8}N_{mb}CP-\eta _{9}N_{a}P-qN_{a}, \end{aligned}$$

where $f_{1}(t)$ takes the functional form given in Fig. 2a so that the available macro and micronutrients decrease by 20% from age 20 to age 80 years.

It has been shown in^47,48^, and references therein, that using faecal samples as surrogates of intestinal microbiota composition demonstrates that this composition differs between young and older individuals, with the latter having increased facultative anaerobes (opportunistic proinflammatory bacteria) and also higher plasma levels of pro-inflammatory mediators. Microbial imbalance has also been associated with low levels of short chain fatty acids (SCFAs)^47^. With advancing age, macrophages are less effective at clearing pathogenic bacteria^47,49^ ($\beta _{4}$ decreases) and there is an age-dependent decline in autophagy ($\beta _{3}$ decreases) that promotes pathological ageing and disease^50^. The production of SCFAs and anti-microbial proteins (AMPs) by commensal microbes that directly inhibit the growth of pathogens also declines ($\beta _{5}$ decreases), in addition to a decrease in the expression of molecules involved in immunoglobulin production^48^ by B-cells ($\beta _{6}$ decreases) that reduce secretory IgA in the mucus layer. The responses of sIgA to pathogens also decreases^43^, and hence the subsequent elimination of pathogens by innate immune cells such as macrophages is downregulated.

The rates of intake ($F^{0}$, $P^{0}$), proliferation ($\beta _{1}\eta _{3}$, $\beta _{1}\eta _{7}$, $\beta _{2}\eta _{4}$, $\beta _{2}\eta _{9}$) and faecal removal (*q*) of commensal and pathogenic bacteria are assumed to remain constant with increasing age.

The equations governing the number of bacteria in the commensal (*F*) and pathogenic (*P*) populations then satisfy

7 $$\begin{aligned} \frac{dF}{dt}= & F^{0}+P_{b}+f(B_{T})\beta _{1}(\eta _{3}N_{mi}+\eta _{7}N_{mb})F-qF, \end{aligned}$$

8 $$\begin{aligned} \frac{dP}{dt}= & P^{0}+f(B_{T})\beta _{2}(\eta _{4}N_{mi}+\eta _{9}N_{a})P-f_{2}(t)\left( \beta _{3}E+\beta _{4}M+\beta _{5}F+\beta _{6}B\right) P \nonumber \\ & -qP, \end{aligned}$$

where $f_{2}(t)$ is a decreasing function with age and takes the form given in Fig. 2b. Probiotic supplementation, for example of one of the *Lactobacillus* strains mentioned in the Introduction, is incorporated by the input term for the commensal bacteria population at rate $P_{b}$ so here, *Lactobacillus* can be viewed as shorthand for the commensal population. A schematic summary of the interactions between the nutrients and microbiota is provided in Fig. S2.

At age 20 years (representing a healthy adult) the microbiota are assumed to be in homeostasis so that at $t=0$

9 $$\begin{aligned} & N_{ma_{0}}=N_{ma_{ss}}, \ \ N_{mi_{0}}=N_{mi_{ss}}, \ \ N_{mb_{0}}=N_{mb_{ss}}, \nonumber \\ & N_{a_{0}}=N_{a_{ss}}, \ \ F_{0}=F_{ss}, \ \ P_{0}=P_{ss} \end{aligned}$$

where subscript _ss_ denotes the nutrient concentrations and bacteria populations at the steady state.

#### Parameter values

The derivation of the parameter values is given in^36^ (wherein the parameter values are all held constant), along with the local sensitivity of the model to each parameter. For ease, values are reproduced here in Table 1. Limited information is available about a number of the parameters (both here and in the subsequent subsections) and it should be emphasised that a central goal of the analysis in this paper is to gain insights into qualitative trends, rather than for the most part to obtain detailed quantitative predictions.

### Vitamin D and the vitamin D receptor

Older adults are regarded as an at-risk population for vitamin D deficiency due to age-related changes in vitamin D metabolism and lifestyle factors^51^. In the absence of active supplementation, the majority of vitamin D is generated by sunlight on skin (approximately 90%, depending upon season). However, there is a decrease in the concentration of 7-dehydrocholesterol in the epidermis and a reduced response to UV light in old compared with young individuals, resulting in a 50% decrease in the formation of vitamin D in older people. In^51,52,^ an age-related reduction in vitamin D production of 13% per decade was calculated, also demonstrating production at 70 years to be half of that at 20 years. In^53^, measurements of serum 25(OH)D in 188 and 122 Chinese women and men, respectively, showed approximately a 20% decrease in serum 25(OH)D levels in men between 20 and 80 years. Other factors that influence vitamin D levels in the ageing population e.g. smoking, BMI and sex are discussed in^54^. In^55^ production of 1,25(OH)_2_D reduced by 50% because of age-related decline in renal function. As 1,25(OH)_2_D is dependent upon an adequate supply of the substrate 25(OH)D, the development of vitamin D (25(OH)D) deficiency leads to further reduction in the formation of 1,25(OH)_2_D.

It is likely that most of the immune functions of vitamin D occur via an intracrine (localised activation and modulation of the vitamin D receptor, VDR) mechanism rather than a systemic one. There are no data for age-related changes in immune cell conversion of 25(OH)D to 1,25(OH)_2_D and this is likely to be very different from the endocrine renal metabolic conversion (where physical decline in renal function impacts circulating levels of 1,25(OH)_2_D). There is also likely to be a decrease in outer cell membrane integrity of macrophages and epithelial cells with increasing age. Additionally^56^, found that binding of 1,25(OH)_2_D to the VDR is reduced in old avian and mammalian intestinal cells.

The rates of generation and metabolism of 25(OH)D ($D^{0}$, $D^{1}$ and $k_{d}$, respectively) and the binding of 1,25(OH)_2_D to the vitamin D receptor ($\delta _{6}$) are therefore assumed to decline with age and the permeabilities of the macrophages and epithelial cells to free 25(OH)D and 1,25(OH)_2_D ($\sigma _{1}$ and $\sigma _{2}$, respectively) increase with age, while the metabolism of intracellular 25(OH)D to 1,25(OH)_2_D ($k_{d_{i}}$) declines. Probiotic supplements increase serum concentrations of 25(OH)D and can increase intestinal vitamin D absorption^57^. However^41^, determined that ageing does not affect the ability of probiotics to elicit protective epithelial innate immunity. All other rates of degradation ($\delta _{2}$, $\delta _{3}$, $\delta _{4}$, $\delta _{5}$ and $\delta _{7}$) and Michaelis-Menten constants ($K_{D}$, $K_{D_{i}}$) are taken to be independent of age. In contrast to^36^, the availability of 25(OH)D from dietary sources (which includes supplements) has been separated from that generated via sunlight as the age-related rate changes differ between each source i.e. the rate reduction in generation by the skin with age is likely to be greater than that by nutrient absorption in the gut. $D^{0}$ now represents the availability of 25(OH)D from sunlight and an additional term $D^{1}$ which denotes the availability of 25(OH)D from dietary sources has been included. As approximately 90% of vitamin D is generated by sunlight it is assumed that $D^{0}=9D^{1}$. The equations governing the concentration of extracellular 25(OH)D (*D*) and 1,25(OH)_2_D ($D_{a}$), intracellular 25(OH)D ($D_{i}$) and 1,25(OH)_2_D ($D_{a_{i}}$) and VDR:1,25(OH)_2_D complex ($V_{D_{a}}$) are then given by

10 $$\begin{aligned} \frac{dD}{dt}= & (f_{3}(t)D^{0}+f_{1}(t)D^{1})\left( 1+\frac{\delta _{1}P_{b}}{K_{\delta }+P_{b}}\right) -\frac{f_{4}(t)k_{d} D}{\delta (1+D_{a})(K_{D}+D)}-\delta _{2}D, \end{aligned}$$

11 $$\begin{aligned} \frac{dD_{a}}{dt}= & \frac{f_{4}(t)k_{d} D}{\delta (1+D_{a})(K_{D}+D)}-\delta _{3}D_{a}, \end{aligned}$$

12 $$\begin{aligned} \frac{dD_{i}}{dt}= & (\mu _{f}D-D_{i})f_{5}(t)(\sigma _{1}M+\sigma _{2}E)-\frac{f_{6}(t)k_{d_{i}} D_{i}}{\delta _{i}(1+D_{a_{i}})(K_{D_{i}}+D_{i})}-\delta _{4}D_{i}, \end{aligned}$$

13 $$\begin{aligned} \frac{dD_{a_{i}}}{dt}= & (\mu _{f_{a}}D_{a}-D_{a_{i}})f_{5}(t)(\sigma _{1}M+\sigma _{2}E)+\frac{f_{6}(t)k_{d_{i}} D_{i}}{\delta _{i}(1+D_{a_{i}})(K_{D_{i}}+D_{i})}-\delta _{5}D_{a_{i}}, \end{aligned}$$

14 $$\begin{aligned} \frac{dV_{D_{a}}}{dt}= & \frac{f_{7}(t)\delta _{6}\delta _{8}(a+P_{b})D_{a_{i}}}{P_{b}+K_{V}}-\delta _{7}V_{D_{a}}, \end{aligned}$$

where $f_{3}(t)$, $f_{4}(t)$, $f_{6}(t)$ and $f_{7(t)}$ are decreasing functions with age and $f_{5}(t)$ is an increasing function. They take the forms given in Fig. 3a-c and $f_{1}(t)$, the age-related reduction in nutrient absorption, is shown in Fig. 2a. A 13% age reduction per decade has been adopted in the production of 25(OH)D ($f_{3}(t)$) between the ages of 20 and 80 years and a corresponding 50% reduction in production of 1,25(OH)_2_D ($f_{4}(t)$) over this time frame as suggested in^51,55^. The permeability of the macrophage and epithelial cell membrane is assumed to increase by 20% ($f_{5}(t)$) and the rate of metabolism of intracellular 25(OH)D to 1,25(OH)_2_D decrease by 20% ($f_{6}(t)$). The sensitivity analysis presented in^36^ shows that the model is insensitive to the parameters $\sigma _{1}$, $\sigma _{2}$ and $k_{d_{i}}$, so the age-related changes represented by $f_{5}(t)$ and $f_{6}(t)$ can be chosen arbitrarily. A 20% decline in the binding of VDR to 1,25(OH)_2_D has also been assumed between the young and older adults ($f_{7}(t)$). A schematic summary of the metabolism of 25(OH)D is provided in Fig. S3.

At age 20 years, the concentration of serum 25(OH)D is assumed to be constant and the concentration of its metabolites are at steady state, so that at $t=0$

15 $$\begin{aligned} & D_{0}=D_{ss}, \ \ D_{a_{0}}=D_{a_{ss}}, \ \ D_{i_{0}}=D_{i_{ss}}, \ \ D_{ai_{0}}=D_{ai_{ss}}, \ \ V_{D_{a_{0}}}=V_{D_{a_{ss}}}. \end{aligned}$$

#### Parameter values

Parameter values for the vitamin D and VDR model are taken from^36^ and shown in Table 2.

### The intestinal epithelial barrier and the immune response

Studies in aged humans have found that the composition of the gut microbiome changes with age. Microbial imbalance is associated with high levels of pro-inflammatory cytokines and low levels of SCFA production, disrupting the stability of the intestinal epithelial tight junctions^42^. The make-up of the epithelium also changes with age, particularly for Paneth and Goblet cells, which appear to decrease with age and are the source of AMPs and mucus, respectively^59^. The mucus barrier can become thinner, impacting barrier function and mucus adherent bacteria^60^. The adherence of health-promoting Bifidobacterium to mucus decreases with age in humans, which is mirrored by its reduced abundance in the microbiota of aged individuals^61^. The resulting increase in gut permeability allows pathogens and their products to translocate into the systemic circulation^47^.

Studies suggest that intestinal stem cell numbers increase in the aged intestinal mucosa, yet there is a decrease in crypt cell numbers. This may be explained by decreased cell division and/or survival with age and lower rates of division, related to alterations in intracellular signalling pathways. By binding to the VDR, the active hormone 1,25-dihydroxyvitamin D (1,25 (OH)_2_D) influences signalling pathways that regulate cell proliferation, differentiation, and cell survival^62^. In humans, the permeability of the intestinal epithelium to macromolecules is not affected by ageing but it may be more permeable to solutes, meaning that any increase in intestinal permeability is not due to disturbances to the overall morphology of tight junction^41^ but instead, due to increased IL-6 levels having an impact on the expression of the tight junction protein Claudin-2, with direct bearing on the intestinal permeability^41^. Levels of IL-6 (pro-inflammatory) have been shown to increase in older adults and dendritic cells and macrophages play an important part in this. The levels of Claudin-2 were observed to increase significantly in the ageing group studied in^41^.

As humans age, macrophages become less effective at clearing damaged epithelial cells and stimulating tissue regeneration^47,49^. Additionally, the mucosal immune system becomes less effective at protecting the healthy epithelial cells from pathogens due to the decrease in production of secretory IgA.

The rate of proliferation of epithelial cells ($\epsilon _{1}$), rate of repair of damaged epithelial cells by VDR ($\epsilon _{2}$) and the rate of removal of damaged epithelial cells by macrophages ($\epsilon _{3}$) are therefore assumed to all decline with age. Moreover, the damage to epithelial cells by pro-inflammatory mediators ($\epsilon _{4}$) and pathogenic bacteria ($\epsilon _{5}$) increases with age. The equations governing the volume fractions of healthy (*E*) and damaged ($E_{d}$) epithelial cells then become

16 $$\begin{aligned} \frac{dE}{dt}= & f_{8}(t)\left( \epsilon _{1}N_{mb}+(\epsilon _{2}V_{D_{a}}+\epsilon _{3}M)\right) E_{d}-f_{9}(t)(\epsilon _{4}C+\epsilon _{5}P)E, \end{aligned}$$

17 $$\begin{aligned} \frac{dE_{d}}{dt}= & f_{9}(t)(\epsilon _{4}C+\epsilon _{5}P)E-f_{8}(t)\left( (\epsilon _{2}V_{D_{a}}+\epsilon _{3}M)+ \epsilon _{1}N_{mb}\right) E_{d}, \end{aligned}$$

where $f_{8}(t)$ and $f_{9}(t)$ are decreasing and increasing functions of age, respectively in the forms shown in Fig. 4a and 4b.

In addition to decreased phagocytosis and immune resolution, macrophages exhibit increased senescence-associated markers and increased basal inflammatory cytokine production with age^47,49^. However, the migration of macrophages (and neutrophils) towards high concentrations of inflammatory cytokines declines after the age of 30 years, reducing to approximately one third by age 70 years^37^ (see Fig. 6c).

The T-cell pool contains several well-defined, functionally distinct subsets, each of these being further divided into subpopulations (such as Th1, Th2, Th17 and regulatory T-cells (Tregs)). While not all T-cell compartments are equally affected by age, overall T cell numbers decline with age as thymic involution leads to decreased output of new/naïve T-cells^38,48,63^. Figure 6a and b indicate that changes to the thymic output of T-cells occur from late teens/early twenties, declining to less than 10% of its peak value by the age of 80 years. There is a similar decrease in magnitude of T-cell output between men and women. However, profiles differ with T-cell outputs plateauing between 40 and 55 years in women before declining again in later years. With dwindling thymic T-cell production, homeostatic proliferation of peripheral T-cells has to compensate and is responsible for maintaining naïve T-cell numbers. While this is an effective mechanism, it eventually fails, ultimately resulting in a decrease in the total number of naïve T-cells and allowing memory and effector T-cells to become dominant in trying to maintain T cell numbers. It has also been proposed that age-related deviations in immune competence may partially reflect changing function in T regulatory cells, profoundly affecting the balance between protective and pathogenic immune responses^63^.

Naturally occurring Tregs (nTreg) seem to accumulate with advancing age but induced Treg cells (iTreg) decrease^63,64^. It is thought that an increase in Treg numbers leads to impaired anti-pathogen responses and may contribute to the high risk of disease reactivation, such as shingles, typically encountered in individuals older than 60 years of age. Given the critical role of Tregs in immune homeostasis, any decline in Treg competence would inevitably lead to a disbalance in protective and pathogenic immunity and would favour chronic relentless and possibly tissue-damaging inflammation.

Ageing decreases B-cell differentiation in the bone marrow, as well as the output of mature B-cells able to make antibodies, inducing a redistribution of B-cell subsets in the periphery with a significant increase in numbers of pro-inflammatory B-cells and increased clonality of antibody responses^48^. The diversity of antibody repertoires that B-cells make is therefore more restricted and their affinity reduces so they become less functional with age.

To summarise, the rate of activation of macrophages by pro-inflammatory cytokines ($\iota _{1}$) and the production of anti-inflammatory mediators by macrophages ($\alpha _{4}$) are assumed to decline with age. The proliferation and activation of antigen-specific T-cells in response to metabolites ($\iota _{8}$), pro-inflammatory cytokines ($\iota _{9}$) and pathogenic bacteria ($\iota _{10}$) also reduces with age, along with the activation and maturation of B cells required to make antibodies ($\iota _{14}$, $\iota _{15}$). However, the production of pro-inflammatory cytokines by macrophages ($\alpha _{7}$) and T-cells ($\alpha _{10}$) increases with age as they become more inflammatory. Also, the natural death rate of immune cells ($\iota _{3}$, $\iota _{7}$, $\iota _{13}$ and $\iota _{17}$) and degradation of cytokines ($\alpha _{5}$, $\alpha _{11}$), the rate of proliferation of regulatory cells ($\iota _{4}$, $\iota _{5}$), the rate of inhibition of macrophages by anti-inflammatory mediators ($\iota _{2}$), the production of anti-inflammatory cytokines by regulatory cells and epithelial cells ($\alpha _{2}$, $\alpha _{g}$ and $\alpha _{1}$) and the production of pro-inflammatory cytokines by damaged epithelial cells ($\alpha _{c}$ and $\alpha _{6}$) are all assumed to be independent of age. The equations governing the densities of macrophages (*M*), regulatory cells (*R*), T-cells ($T_{h}$) and B-cells (*B*), and concentrations of anti- (*G*) and pro-inflammatory (*C*) cytokines are then given by

18 $$\begin{aligned} \frac{dM}{dt}= & f_{10}(t)\iota _{1}C-\iota _{2}GM -\iota _{3}M, \end{aligned}$$

19 $$\begin{aligned} \frac{dR}{dt}= & \frac{(\iota _{4}V_{D_{a}}+\iota _{5}F)}{1+\iota _{6}P}-\iota _{7}R, \end{aligned}$$

20 $$\begin{aligned} \frac{dT_{h}}{dt}= & f_{11}(t)\frac{(\iota _{8}N_{mb}+\iota _{9}C+\iota _{10}P)}{(1+\iota _{11}V_{D_{a}}+\iota _{12}F)}-\iota _{13}T_{h}, \end{aligned}$$

21 $$\begin{aligned} \frac{dB}{dt}= & f_{12}(t)\frac{\iota _{14}N_{mb} + \iota _{15}C}{1+\iota _{16}V_{D_{a}}}-\iota _{17}B, \end{aligned}$$

22 $$\begin{aligned} \frac{dG}{dt}= & \gamma _{g}+(\alpha _{g}+\alpha _{1}V_{D_{a}})E+\alpha _{2}FR+f_{13}(t)(\alpha _{3}E_{d}+\alpha _{4}P)M-\alpha _{5}G, \end{aligned}$$

23 $$\begin{aligned} \frac{dC}{dt}= & \gamma _{c}+(\alpha _{c}+\alpha _{6}P)E_{d}+f_{14}(t)\frac{\alpha _{7}M}{(1+\alpha _{8}V_{D_{a}}+\alpha _{9}F)}+f_{15}(t)\alpha _{10}PT_{h}-\alpha _{11}C \end{aligned}$$

Here $f_{10}(t)$, $f_{11}(t)$, $f_{12}(t)$ and $f_{13}(t)$ are decreasing and $f_{14}(t)$ and $f_{15}(t)$ increasing functions of age. $f_{10}(t)$ and $f_{11}(t)$ take the forms shown in Fig. 6a and c. For simplicity, the profile for men for $f_{10}(t)$ is adopted here as it depicts a continuous decline with age. Similar functional forms for $f_{12}(t)$ and $f_{13}(t)$ are assumed and the mirror image of $f_{11}(t)$ for $f_{14}(t)$ and $f_{15}(t)$ as shown in Fig. 5a–c. A schematic summary of the interactions between the epithelial barrier and immune response is provided in Fig. S4.

Initial conditions at age 20 years for the immune system at $t=0$ are

24 $$\begin{aligned} & E_{0}=E_{ss}, \ \ E_{d_{0}}=E_{d_{ss}}, \ \ M_{0}=M_{ss}, \ \ R_{0}=R_{ss} \nonumber \\ & T_{h_{0}}=T_{h_{ss}}, \ \ B_{0}=B_{ss}, \ \ G_{0}=G_{ss}, \ \ C_{0}=C_{ss}. \end{aligned}$$

#### Parameter values

Parameter values for the intestinal epithelial barrier and immune response model are taken from^36^ and are given in Table 3.

### Global sensitivity analysis

The majority of the parameters (i.e. those without literature references) in Tables 1, 2 and 3 are adopted on plausibility grounds. To identify parameters that most strongly influence model outcomes, a global sensitivity analysis is performed on these uncertain parameters. We solve our system of ODEs, drawing a Latin Hypercube sample from the range of values determined by $\pm 50\%$ of the stated value using the function randomLHS in *R*. For each sampled parameter set, the model is simulated to steady state and the final values of the model output variables extracted. Partial Rank Correlation Coefficients (PRCCs) are then calculated using the pcc function in *R* to quantify the influence of each parameter on model outputs, allowing a ranking of parameters by their relative impact.

## Supplementary Information

Supplementary Information.

## Data Availability

The datasets used and/or analysed during the current study available from the corresponding author on reasonable request.
