# Supplementary material for: Modelling the influence of vitamin D and probiotics on inflammation and the intestinal microbiota in older adults
Source: Sci Rep. 2025 Nov 26;15:42048. doi: 10.1038/s41598-025-26132-8 (PMC12657869; doi:10.1038/s41598-025-26132-8)
Supplement: Supplementary file 1 — Supplementary Information. [file 41598_2025_26132_MOESM1_ESM.pdf]

Supplementary Information for Modelling the influence of vitamin D and probiotics on inflammation and the intestinal microbiota in older adults

S.J. Franks[1], J.L. Dunster[1,2], S.R. Carding[3,4], J.M. Lord[5], M. Hewison[6], P.C. Calder[7,8], J.R. King[1]

[1]School of Mathematical Sciences, University of Nottingham, Nottingham, UK
[2]Institute for Cardiovascular and Metabolic Research, University of Reading, Reading UK
[3]Quadram Institute Biosciences, Norwich Research Park, Norwich, UK
[4]Norwich Medical School, University East Anglia, Norwich, UK
[5]Institute of Inflammation and Ageing, University of Birmingham, Birmingham, UK
[6]Institute of Metabolism and Systems Research, University of Birmingham, Birmingham, UK
[7]School of Human Development and Health, Faculty of Medicine, University of Southampton, Southampton, UK
[8] NIHR Southampton Biomedical Research Centre, University Hospital Southampton NHS Foundation Trust and University of Southampton, Southampton, UK

This pdf includes:

- Supplementary text
- Supplementary figures S1-S4

Supplementary text and figures

Discussion on using the LSODA solver

The LSODA solver employs adaptive step-size control to balance efficiency and accuracy during integration. When system dynamics are smooth, the solver lengthens the step size to reduce computational burden, while in regions of rapid change, steps are shortened to maintain error control. A key strength of LSODA is its automatic detection of numerical stiffness and its ability to switch between non-stiff (Adams) and stiff (BDF) methods, allowing stable integration of systems that couple slow and fast processes. Over long simulation horizons, however, even adaptive solvers can accumulate truncation and rounding errors, which may influence the stability of equilibria or lead to slight drift in long-term behaviour. It is therefore advisable to confirm equilibrium results by testing solver tolerances or restarting from candidate steady states. Finally, while LSODA is available across platforms and languages, reproducibility may not be exact due to differences in floating-point arithmetic, compiler behaviour, and default solver settings. For this reason, reproducibility is best ensured by documenting solver tolerances, initial and maximum step sizes, and by comparing qualitative features of the dynamics—such as equilibrium states or oscillatory patterns—rather than expecting identical numerical trajectories.

S1

The schematic shown in Figure S1 summarises the complex interactions between the three sub-models i.e. the intestinal microbiota, vitamin D and the immune response captured by the model.

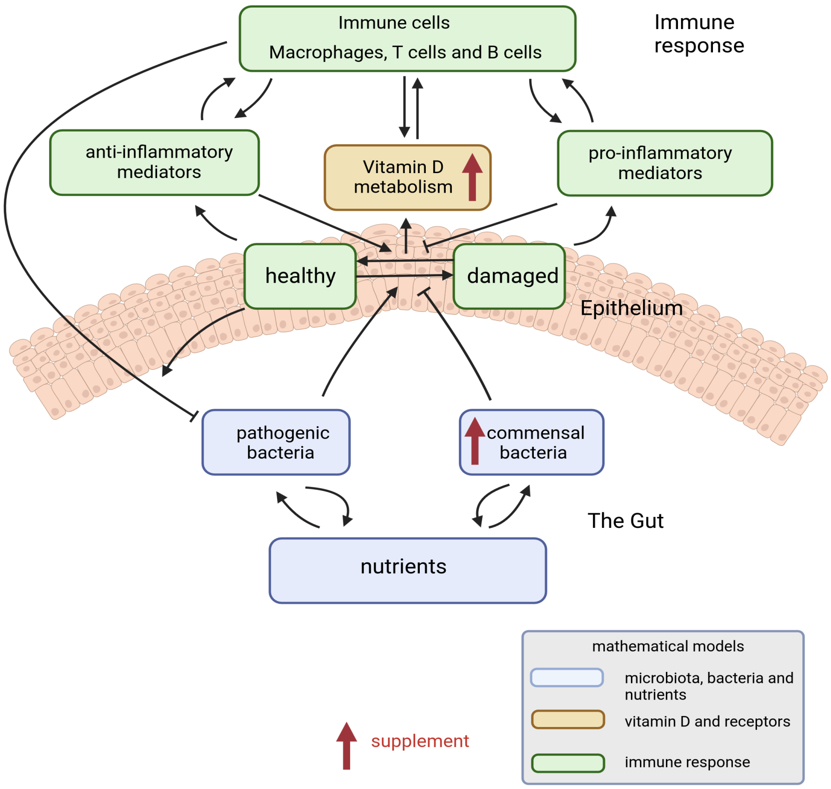

Fig. S1: The interactions between the microbiota, vitamin D and the immune response captured in the mathematical models presented the Methods section

S2

Figure S2 summarises the interactions between the microbiota and nutrient environment described by Eqns (3)-(8).

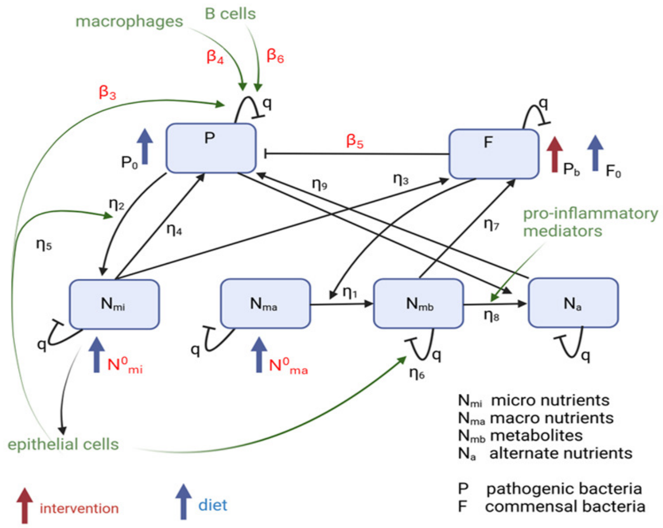

Fig. S2: **The microbiota and nutrient network.** The model derived in Eqns (3)-(8) captures the reactions between commensal and pathogenic bacteria, macronutrients, micronutrients, metabolites and alternate nutrients. The rates are defined in Table 1 and those in red are age-dependent.

S3

Figure S3 illustrates the metabolism of 25(OH)D described by Eqns (10)-(14).

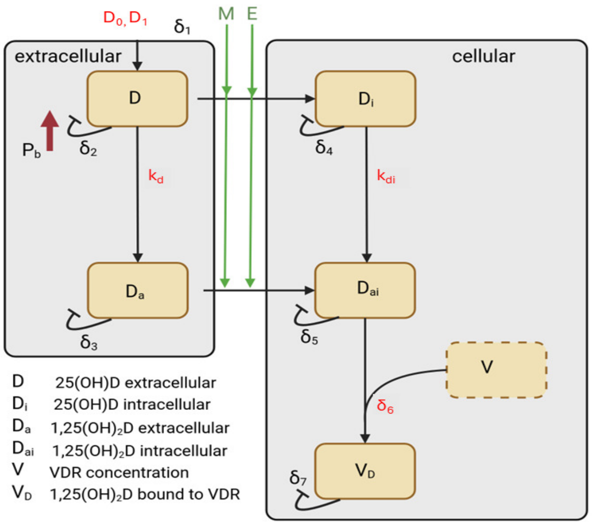

Fig. S3: **The vitamin D network.** The model derived in Eqns (10)-(14) describes the conversion of 25(OH)D into its active form 1,25$(OH)_2$D, the diffusion of the free forms of these across the epithelial and macrophage cell membranes and the binding with the vitamin D receptor. The rates are defined in Table 2 and those in red are age-dependent.

S4

Figure S4 summarises the interactions between the intestinal epithelial barrier and the immune system described by Eqns (16)-(23).

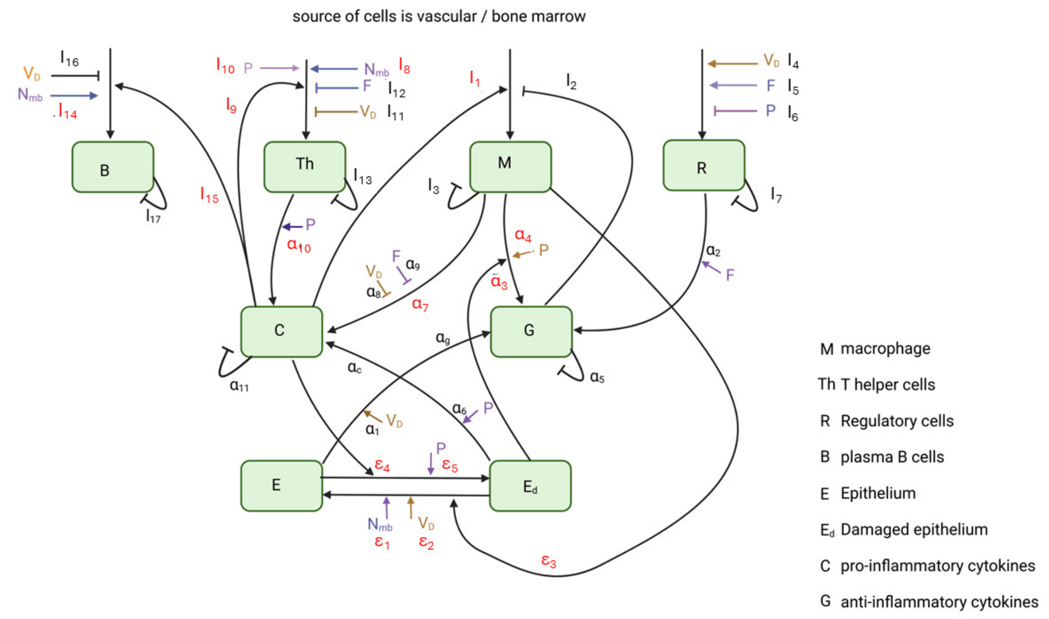

Fig. S4: **The immune response network.** The model derived in equations (16)-(23) details the interactions between the intestinal epithelial barrier and the innate and adaptive immune responses. The parameters are defined in Table 3 and those in red are age-dependent.
